# Partitioning and Mobility of Chromium in Iron-Rich Laterites from an Optimized Sequential Extraction Procedure

**DOI:** 10.1021/acs.est.3c10774

**Published:** 2024-03-29

**Authors:** Ruth Esther G. Delina, Jeffrey Paulo H. Perez, Jessica A. Stammeier, Elena F. Bazarkina, Liane G. Benning

**Affiliations:** † GFZ German Research Centre for Geosciences, Telegrafenberg, 14473 Potsdam, Germany; ‡ Department of Earth Sciences, Freie Universität Berlin, 12249 Berlin, Germany; § The Rossendorf Beamline at ESRF, The European Synchrotron, CS 40220, 38043 Grenoble Cedex 9, France; ∥ Institute of Resource Ecology, Helmholtz-Zentrum Dresden-Rossendorf, Bautzner Landstraβe 400, 01328 Dresden, Germany

**Keywords:** Cr(VI), dissolution, iron (oxyhydr)oxides, metal substitution, mineral synthesis, nickel laterite, SEP optimization, SEP validation

## Abstract

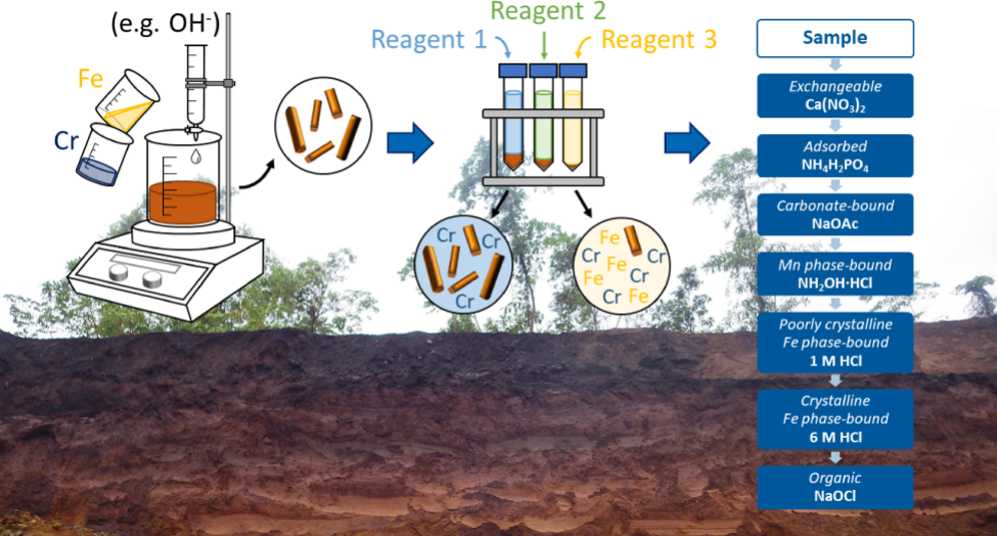

Chromium (Cr) leached from iron (Fe) (oxyhydr)oxide-rich tropical laterites can substantially impact downstream groundwater, ecosystems, and human health. However, its partitioning into mineral hosts, its binding, oxidation state, and potential release are poorly defined. This is in part due to the current lack of well-designed and validated Cr-specific sequential extraction procedures (SEPs) for laterites. To fill this gap, we have (i) first optimized a Cr SEP for Fe (oxyhydr)oxide-rich laterites using synthetic and natural Cr-bearing minerals and laterite references, (ii) used a complementary suite of techniques and critically evaluated existing non-laterite and non-Cr-optimized SEPs, compared to our optimized SEP, and (iii) confirmed the efficiency of our new SEP through analyses of laterites from the Philippines. Our results show that other SEPs inadequately leach Cr host phases and underestimate the Cr fractions. Our SEP recovered up to seven times higher Cr contents because it (a) more efficiently dissolves metal-substituted Fe phases, (b) quantitatively extracts adsorbed Cr, and (c) prevents overestimation of organic Cr in laterites. With this new SEP, we can estimate the mineral-specific Cr fractionation in Fe-rich tropical soils more quantitatively and thus improve our knowledge of the potential environmental impacts of Cr from lateritic areas.

## Abstract 2

We developed a new sequential extraction procedure and optimized the partitioning of chromium in iron (oxyhydr)oxide-rich laterites, allowing us to better evaluate its potential environmental impacts.

## Introduction

Laterite broadly refers to the iron (Fe) or aluminum (Al) (oxyhydr)oxide-rich weathering mantle covering about 33% of the continents.^1,2^ Laterites developed from tropical weathering of ultramafic rocks (e.g., peridotites and dunites) predominantly consist of Fe (oxyhydr)oxides in the form of goethite (FeOOH) and hematite (Fe_2_O_3_) and are often enriched with critical metals such as nickel (Ni), cobalt (Co), and scandium (Sc) mainly incorporated into the minerals.^3−5^ Such metal deposits are known as nickel laterites, and they are the world’s main source of Ni, accounting for ∼60% of the global production.^4,6^

Nickel laterites also contain elevated concentrations of chromium (up to ∼70,000 mg kg^–1^)^7,8^ that are multiple orders of magnitude higher than upper crustal averages (35 mg kg^–1^).^9^ Chromium commonly occurs as Cr(III) and Cr(VI), with the latter being a highly mobile, toxic, and carcinogenic pollutant.^10−12^ The majority of Cr in Ni laterites is present as Cr(III) and preferentially substitutes into octahedral sites of Fe (oxyhydr)oxides (e.g., goethite and hematite), silicates, and spinels,^7,13^ while Cr(VI) predominantly exists as oxyanions (i.e., HCrO_4_^–^ and CrO_4_^2–^) adsorbed onto these same minerals^14,15^ or dissolved in pore waters or soil solutions.^16^ Since Ni laterites are exploited through large opencast surface mining, Cr(VI) leaches into surface- and groundwaters where it can reach concentrations (up to 1600 μg L^–1^),^17−19^ far exceeding international drinking water standards (50–100 μg L^–1^).^20,21^ Elevated levels of Cr(VI) in these water resources can lead to, so far, not well-understood health issues for the local population.^7^ Thus, it is important to quantify the partitioning and possible transport mechanisms of Cr species in laterite host phases and evaluate how these Cr-mineral phase specific associations dictate the potential mobility, bioavailability, and toxicity of Cr during geogenic (e.g., weathering) and anthropogenic (e.g., mining) processes.

The partitioning of elements in soils and sediments is traditionally evaluated through sequential extraction procedures (SEPs), which are based on a series of increasingly aggressive reagents that categorize the leached elements into chemical or mineralogical fractions.^22,23^ However, SEPs are criticized for poor selectivity of extraction reagents, redistribution of metals, and incomplete dissolution.^22,24−26^ For instance, in the case of Cr, most SEPs cannot completely dissolve common host phases such as chromite and Fe (oxyhydr)oxides.^18,27^ Chromites are highly recalcitrant to dissolution with most conventional digestion methods,^18,24,28^ while the dissolution of Fe (oxyhydr)oxide is known to be affected by metal substitution. For example, substitution of Cr and Al for Fe in goethite has been shown to strongly inhibit its dissolution in strong acids and reductants;^23,29^ yet the effect of metal substitution is often overlooked when developing SEPs. In addition, SEPs are commonly optimized for cationic species,^22^ and thus when applied to Cr, they likely underestimate the distribution of Cr(VI) oxyanions. More importantly, no SEP has been critically assessed for its suitability for Cr partitioning in tropical laterites, which possess such a unique Fe mineral assemblage. Existing SEPs applied for Cr fractionation in tropical soils rich in Fe and Mn (oxyhydr)oxides^18,28,30,31^ were originally developed for other metals and/or sample matrices. These include the modified Geological Survey of Canada (mGSC) procedure, which was initially developed to partition Cd in temperate soils^32^ but has also been tested to be suitable for tropical soils.^33^ The SEP used in Quantin et al.^28^ was adapted from well-cited procedures, including Tessier et al.,^34^ which were intended for extracting metals such as Si, Ca, Cd, and Fe from river sediments and temperate to subtropical soils.^34−36^ Finally, the SEP by Silveira et al.^23^ was designed for tropical soils and optimized for Zn, Cu, Fe, and Mn but not Cr. Because these SEPs are optimized neither for Fe-rich laterites nor for Cr species, there is a need to optimize a Cr SEP and thus provide a more quantitative evaluation of the fate and potential impacts that Cr can have in such lateritic environments.

To address this gap, we have characterized the partitioning of Cr in various tropical Ni laterite profiles using a new SEP for Fe (oxyhydr)oxide-rich laterites. We optimized different extractants using Cr- and Fe-bearing phases commonly present in Ni laterites and certified laterite references and validated our new SEP using Ni laterites from different localities in the Philippines. We also compared and contrasted our results with the three aforementioned SEPs^23,28,32^ and documented the far more efficient and targeted nature of our new SEP.

## Materials and Methods

### Natural Laterites

The partitioning of Cr was examined in previously well-characterized Ni laterites from three major Ni mining districts in the Philippines (Palawan,^18^ Zambales, and Surigao^37^). Palawan samples described in Delina et al.^18^ were obtained from a 6.8 m thick Ni laterite profile consisting of an upper Fe (oxyhydr)oxide (i.e., goethite and hematite) dominated limonite zone and a lower silicate-rich (i.e., serpentine and smectite) saprolite layer separated by a thin transition zone. From bottom to top, the profile is characterized by a dramatic increase in Cr and Fe contents (from 0.5 to 2.9 wt % Cr and 9 to 54 wt % Fe).^18^ Samples from the limonite, transition, and saprolite zones (hereafter referred to as PAL-1, PAL-2, and PAL-3, respectively), representative of different Cr and Fe concentrations, were used to evaluate the efficiency of our new SEP. Furthermore, the robustness of our SEP was tested on five high Cr (1.1–1.7 wt %) and Fe (38–55 wt %) limonite samples from Zambales (ZAM-1 to ZAM-3) and Surigao (SUR-1 to SUR-2). These primarily contain goethite (>89%) with minor spinel (2.4–11%). Characterization of these samples is discussed in Text S1.

### Synthesis and Preparation of Mineral Standards

Various synthetic and natural mineral references (Table S1) representing the composition of Fe (oxyhydr)oxide-rich laterites^3,18,38^ were prepared to optimize the SEP. Pure and metal (Me)-substituted (Me = Al and Cr) ferrihydrite, goethite (α-FeOOH), hematite (α-Fe_2_O_3_), and pure magnetite [Fe(II)Fe(III)_2_O_4_] were synthesized using standard procedures adapted from Schwertmann and Cornell.^39^ In addition to Cr, Al-substituted Fe minerals were also prepared since pedogenic Fe (oxyhydr)oxides often structurally incorporate Al.^29^ Cr(VI)-adsorbed Fe (oxyhydr)oxides were also prepared. Details of the preparation and characterization of these synthetic minerals and natural samples (e.g., chromite) can be found in Text S2.

### Testing and Optimization Based on the Mineral References

Single extractions (detailed in Text S4) were carried out to assess the dissolution efficiency and selectivity of different reagents. Selection of extractants were based on extensive reviews of SEPs^22,25,40^ and procedures applied to Fe (oxyhydr)oxides and Fe-rich soils and sediments.^23,41−43^ Operating conditions (e.g., temperature, duration, and solid-to-liquid ratio) and concentrations were varied and tested to find the best possible extractant (Tables 1 and S3).

**Table 1:** Dissolution Efficiencies of Single Extractions on Selected Mineral Standards and Sequential Extractions on Mixtures and Laterite CRMs^a^

extractant	ferrihydrite	goethite	hematite	magnetite (synthetic)	magnetite (natural)	chromite
	poorly cryst. Fe Ox	crystalline Fe Ox				residual
	Fe dissolution efficiency (%)					
0.1 M Ca(NO_3_)_2_	*bdl*	*bdl*	*bdl*	*bdl*	*bdl*	*bdl*
0.01 M NH_4_H_2_PO_4_	0.02 (8 × 10^–4^)	*bdl*	*bdl*	*bdl*	*bdl*	*bdl*
1 M NaOAc	1.40 (0.06)	*bdl*	*bdl*	*bdl*	0.15 (6 × 10^–3^)	*bdl*
0.1 M NH_2_OH·HCl	0.27 (6 × 10^–3^)	*bdl*	0.06 (1 × 10^–3^)	0.11 (2 × 10^–3^)	*bdl*	*bdl*
5% NaOCl (1:20, 2×)	0.004 (1 × 10^–4^)	*bdl*	*bdl*	*bdl*	*bdl*	*bdl*
1 M HCl, 8 h	99.2 (2.3)	0.51 (0.02)	15.4 (0.5)	9.52 (0.32)	0.14 (3 × 10^–3^)	*bdl*
6 M HCl, 75 °C, 24 h	ND	104 (3)	105 (4)	99.0 (3.5)	97.6 (2.6)	*bdl*

extractant	Cr(VI)-ads FHY	Cr(VI)-ads Goe	Cr(VI)-ads Hem	Cr-FHY	Cr-Goe	Cr-Hem	chromite
	adsorbed			poorly cryst. Fe Ox	crystalline Fe Ox		residual
	Cr dissolution efficiency (%)						
0.1 M Ca(NO_3_)_2_	1.20 (0.06)	6.12 (0.31)	7.04 (0.36)	ND	ND	ND	ND
0.01 M NH_4_H_2_PO_4_	76.8 (3.9)	81.3 (4.2)	75.9 (3.9)	ND	ND	ND	ND
5% NaOCl (1:5)	ND	ND	ND	4.07 (0.09)	49.2 (1.4)	4.76 (0.08)	0.15 (6 × 10^–4^)
5% NaOCl (1:20, 2×)	ND	ND	ND	26.0 (1.4)	60.5 (1.7)	15.9 (0.4)	0.12 (1 × 10^–3^)
0.5 M HCl, 4 h	ND	ND	ND	80.6 (2.1)	ND	ND	ND
1 M HCl, 4 h	ND	ND	ND	96.4 (2.5)	ND	ND	ND
1 M HCl, 8 h	ND	ND	ND	98.4 (2.5)	11.6 (0.6)	2.14 (0.07)	*bdl*
6 M HCl, 50 °C, 48 h	ND	ND	ND	ND	52.8 (2.2)	98.6 (5.6)	*bdl*
6 M HCl, 75 °C, 24 h	ND	ND	ND	ND	94.7 (5.7)	102 (6)	*bdl*

extractant	mixture 1	mixture 2	mixture 3	OREAS 182		OREAS 190	
	Cr dissolution efficiency (%)			Cr ext. (%)	Fe ext. (%)	Cr ext. (%)	Fe ext. (%)
step 1: 0.1 M Ca(NO_3_)_2_				0.36 (0.01)	*bdl*	0.04 (1 × 10^–3^)	*bdl*
step 2: 0.01 M NH_4_H_2_PO_4_	51.2 (0.1)			0.47 (0.01)	0.45 (0.01)	0.12 (3 × 10^–3^)	0.26 (6 × 10^–3^)
step 3: 1 M NaOAc				0.01 (2 × 10^–4^)	0.40 (0.01)	0.56 (0.02)	0.37 (8 × 10^–3^)
step 4: 0.1 M NH_2_OH·HCl				0.03 (1 × 10^–3^)	0.23 (5 × 10^–3^)	0.03 (1 × 10^–3^)	0.18 (4 × 10^–3^)
step 5: 1 M HCl, 8 h		102 (2)	89.9 (1.9)	1.01 (0.03)	5.05 (0.11)	0.80 (0.02)	3.94 (0.08)
step 6: 6 M HCl, 75 °C, 24 h	92.0 (1.9)	89.1 (1.9)	108 (2)	11.0 (0.3)	82.7 (1.8)	14.8 (0.4)	75.5 (1.6)
step 7: 5% NaOCl (1:20, 2×)^b^				1.26 (0.04)	*bdl*	0.43 (0.01)	*bdl*
residual	106	109	101				

a FHY—ferrihydrite, Goe—goethite, Hem—hematite, Ox—(oxyhydr)oxides. *Note:* dissolution efficiency or metal extracted (ext.) (%) = (wt % extracted/wt % total) × 100. (#)—analytical uncertainty (<5% relative) based on multiple measurements (*n* ≥ 5) of QC solutions. Mixture compositions are further detailed in Table S5. For mineral mixtures, the residual fraction dissolution efficiency was represented by the (wt % total Cr – ∑wt % non-residual)/wt % Cr in chromite. *bdl*—below detection limit; ND—no data.

b The NaOCl step (step 7) was applied after the 6 M HCl treatment (step 6) to prevent the indiscriminate oxidation of Cr from Fe (oxyhydr)oxides.

To partition adsorbed Cr(VI) oxyanions, we applied an alkaline (pH 8) 0.01 M NH_4_H_2_PO_4_ treatment for 16 h^44,45^ (see Text S3 for detailed information) on Cr(VI)-adsorbed Fe (oxyhydr)oxides. We evaluated the selectivity of typically used extractants for the prior exchangeable fraction step [i.e., 0.1–1 M Ca(NO_3_)_2_ and 1 M MgCl_2_ for 2 h]^25^ with respect to the Cr(VI)-adsorbed phases. We also examined the effect of the following treatments on Cr- and Fe-bearing minerals: 1 M NaOAc buffer (pH 4.5) for 5 h (carbonate-bound fraction),^35,41^ ∼5% NaOCl (pH 8.5) at boiling temperature for 30 min (organic fraction),^46,47^ and 0.1 M NH_2_OH·HCl in 0.01 M HNO_3_ for 10 min (Mn phase-bound fraction).^48^ Furthermore, we assessed the effectiveness of different concentrations of HCl in dissolving Fe (oxyhydr)oxides of different crystallinities. We tested dilute (0.5 and 1 M) HCl^42,49^ for the poorly crystalline fraction and 6 M HCl^23,50^ extractions at different temperatures (50 and 75 °C) and reaction times (≤48 h) for the crystalline fraction.

### Sequential Extractions

Based on the single extractions, we optimized a new SEP and tested it on mixtures of mineral references (Table S5) and Ni laterite certified reference materials (CRMs) (OREAS 182 and 190). The optimized SEP was applied to the Ni laterites and compared to the three SEPs previously used for Cr partitioning in laterites and related tropical soils: the mGSC procedure^32^ (SEP 1), the SEPs used in Quantin et al.^28^ (SEP 2), and Silveira et al.^23^ (SEP 3) (outlined in Table S2). All SEPs were performed in duplicate on the Palawan Ni laterite samples except for SEP 1, which was previously applied to the same samples in Delina et al.^18^

In each SEP step, reagents were mixed with powdered samples in acid-cleaned centrifuge tubes and reacted in temperature-controlled orbital shakers at 150–250 rpm. Liquid phases were separated from the residue by centrifugation at 10,052*g* for 10 min. Between each extraction, residues were washed with Milli-Q water (∼18.2 MΩ·cm) and freeze-dried before the next extraction step. The analysis of the supernatants was identical to that of the single extractions (Text S5). Relative standard deviations (RSDs) of Cr were <5% for ∼80% of samples. RSD >10% was observed in extracts with Cr concentrations near the quantification limit.

The residues of the sequential extraction are chemically resistant minerals such as chromite (see Figure 4) that are highly prone to incomplete dissolution by conventional digestion methods.^18,24,25^ In our work, acid digestion post Na_2_O_2_ fusion^51^ (see Text S1) did not lead to full dissolution, and dark-colored chromite grains persisted. We accounted for the Cr associated with this residual fraction as the difference between the total concentration and the sum of all extracted non-residual fractions, and we mainly discussed and compared steps that target the latter.

To characterize the residual fraction and understand how different SEPs extract Cr, we analyzed the mineralogy and local bonding environment of Cr in selected SEP residues after the crystalline Fe phase-bound step using X-ray diffraction (XRD), scanning electron microscopy (SEM), and high-energy resolution fluorescence detection X-ray absorption spectroscopy (HERFD-XAS), as fully described in the Supporting Information.

## Results and Discussion

### Metal Substitution in Natural Fe (Oxyhydr)oxides

**Figure 1:**
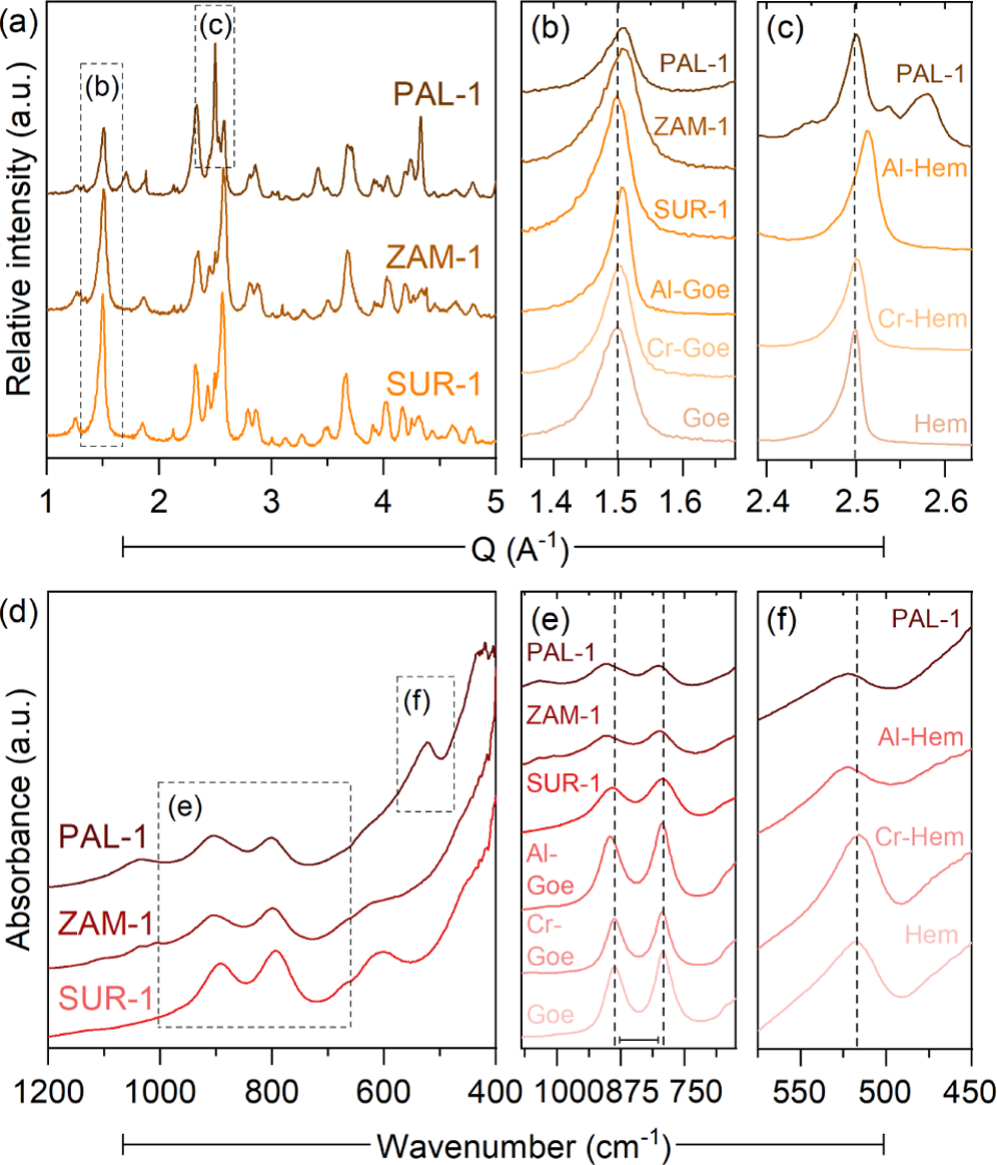
XRD patterns and IR spectra of the Fe (oxyhydr)oxide-rich laterites compared with pure and metal-substituted Fe phases. (a) XRD patterns with highlighted (110) diffraction peaks of (b) goethite (Goe) and (c) hematite (Hem). (d) IR spectra of the samples with highlighted (e) OH bonds of goethite and (f) the Fe–O bond of hematite. Dashed vertical lines highlight peak shifts relative to pure phases.

XRD and infrared (IR) spectroscopy patterns of Fe (oxyhydr)oxide-dominated samples from each Ni laterite district (Figure 1) showed patterns consistent with those of the synthetic goethites (Figures S1 and S2). PAL-1, containing nearly equal amounts of goethite (48%) and hematite (43%), exhibited combined patterns of the Fe phases. Diffraction peaks of the natural samples showed remarkable shifts to higher angles or *Q* (=2π/*d*) values compared with pure goethite (Δ*Q*_(110)_ ≤ 0.012) and hematite (Δ*Q*_(110)_ = 0.003) (Figure 1b,c), suggesting metal substitution. This is supported by the similar shifts displayed by substituted goethites (Δ*Q*_(110)_ ≤ 0.009) and hematites (Δ*Q*_(110)_ ≤ 0.015), indicating a decrease in unit cell volume due to the smaller octahedral radii of Al(III) (0.530 Å, 18% smaller) and Cr(III) (0.615 Å, 5% smaller) compared to Fe(III) (0.645 Å). Consistent with previous studies,^52−55^ Al-substituted phases showed larger shifts due to the significantly smaller atomic radius of Al. The effect of substitution was also observed in the IR spectra (Figure 1e,f), where the separation of the OH bending modes of synthetic goethites at ∼790–890 cm^–1^ increased from 95 to 103 cm^–1^ and the Fe–O band of synthetic hematites at ∼520 cm shifted to higher wavenumbers.

Among the natural goethites, PAL-1 showed the largest diffraction peak shift and IR band separation, which are slightly higher than those of the Al-goethite. This may suggest a higher extent of substitution of many different cations, bigger differences in the atomic radii of substituting metals, or crystal disorder.^52,54−57^ Aside from Al and Cr, Fe (oxyhyr)oxides in laterites have been found to be important hosts for Ni, Co, and Mn.^55,58,59^

### Cr and Fe Extractability from Mineral Standards

Given that Cr in laterites could occur as adsorbed species or structurally incorporated in predominant Fe (oxyhydr)oxides, and metal substitution could affect the crystal structure, and thus, the solubility and dissolution rate of these Fe phases,^59^ we tested the efficiency and selectivity of different extraction steps with a range of Cr and Fe minerals (Tables 1 and S3).

#### Easily Mobilizable Fractions

Our data show that the 0.01 M NH_4_H_2_PO_4_ treatment effectively desorbed more than 70% of Cr from the Cr(VI)-adsorbed Fe (oxyhydr)oxides, with negligible Fe dissolution. A disadvantage of phosphate treatment is that residual adsorbed phosphate can retard Fe dissolution^49,60^ by surface passivation, decreasing the reactivity of the Fe (oxyhydr)oxides.^61−63^ This was evident in the incomplete recovery of Fe from goethite and natural magnetite when the 6 M HCl extraction was preceded by phosphate treatment (Table S4). It is therefore necessary to perform a rinsing step after phosphate extraction. While Ruttenberg^64^ recommended MgCl_2_ wash for phosphorus extractions, we decided to use ultrapure water to minimize dissolved salts in the extract and avoid possible interferences during measurements. A minimum of 3 successive water rinses were found sufficient to displace most of the phosphate (Figure S4).

Ca(NO_3_)_2_ and MgCl_2_ extractions for the exchangeable fraction, usually applied at the beginning of SEPs, indiscriminately extracted up to 20% of Cr from the Cr(VI)-adsorbed Fe (oxyhydr)oxides (Figure S5). To avoid substantial underestimation of adsorbed Cr, the most dilute Ca(NO_3_)_2_ (0.1 M) treatment that extracted only 1–7% of Cr was chosen for the exchangeable fraction. Overall, these experiments imply that previous SEPs without a phosphate step and using only nitrate or chloride salts underestimated the easily mobilizable Cr fraction (exchangeable and adsorbed).

#### Non-Fe Phase-Bound Fractions

Acetate, hypochlorite, and hydroxylamine hydrochloride extractions partitioned very little amounts of Fe (<1%) from the reference phases (Table 1). However, NaOCl leached a significant amount of Cr from all Cr-substituted Fe (oxyhydr)oxides. Among the extractants used for organic matter (e.g., NaOCl, H_2_O_2_, and Na_4_P_2_O_7_), NaOCl was reported to exhibit greater efficiency and minimal attack on amorphous Fe (oxyhydr)oxides and clays in soils.^22,25,65^ However, our results clearly showed that NaOCl treatment leads to substantial Cr release, irrespective of the S/L ratio used. The typical 1:5 ratio^23^ extracted 4–49% of the Cr incorporated in the Fe (oxyhydr)oxides; meanwhile, the 1:20 ratio performed once and twice^46,47^ released 12–56 and 16–60%, respectively. Extracts showed faint to strong yellow hues, suggesting the presence of chromate, and hence, the possible oxidation of Cr(III) to Cr(VI). This aligns with the prior work on the oxidative dissolution of Cr(III) hydroxide with NaOCl.^66,67^ Earlier SEPs of other metals also reported the indiscriminate oxidation of redox-sensitive elements by NaOCl. Gruebel et al.^68^ and Wright et al.^69^ revealed that adsorbed and incorporated Se species in selenides were oxidized to Se(VI), leading to substantial overestimation of the organic pool. Similarly, La Force and Fendorf^46^ showed that Fe(II) from mine wastes were oxidized by NaOCl, resulting in inaccurate partitioning of Fe. Therefore, to avoid the indiscriminate oxidation of Cr, we applied the NaOCl treatment after the Fe (oxyhydr)oxide dissolution.

#### Poorly Crystalline Fe Phase-Bound Fraction

Among the tests using 0.5 and 1 M concentrations and a duration of 4–8 h (Tables 1 and S3), the 8 h 1 M HCl extraction was found to be most effective for poorly crystalline Fe (oxyhydr)oxides. All ferrihydrites were dissolved with >97% total Fe recovery, with Cr-substituted ferrihydrite showing the least recovery. To test the selectivity of 1 M HCl, we applied it to crystalline phases. It extracted up to 15% of total Fe in pure synthetic phases, comparable with earlier works showing dissolution of up to 33% of Fe from synthetic hematite and 9% from magnetite.^42,49^ It should be noted that pure minerals, such as these, rarely occur in nature, and thus, the selectivity of reagents is better evaluated with respect to metal-substituted and natural phases. The substantially low Fe, Cr, and Al dissolution efficiencies (below detection to 12%) (Tables 1 and S3) from metal-substituted and natural Fe (oxyhydr)oxides validate the selectivity of the 1 M HCl treatment.

#### Crystalline Fe Phase-Bound Fraction

**Figure 2:**
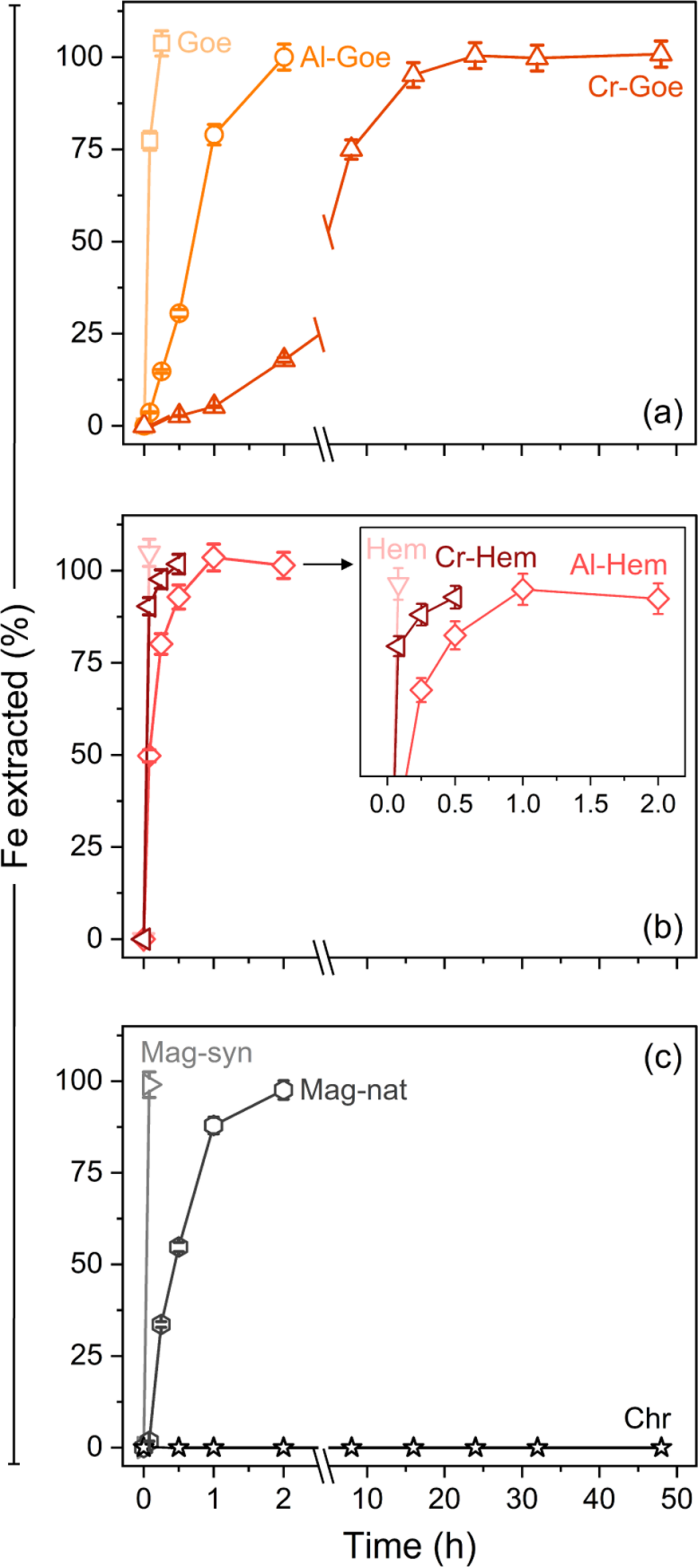
Dissolution time curves of reference Fe minerals: (a) goethites (Goe), (b) hematites (Hem), and (c) spinels (Mag—magnetite, Chr—chromite) in 6 M HCl at 75 °C. Error bars indicate analytical uncertainty (<5% relative) based on multiple measurements (*n* = 8) of QC solutions.

We optimized a 6 M HCl extraction that has been used for the sequential extraction of crystalline Fe oxides and/or sheet silicates.^50,70−72^ Extractions at 50 °C after decreasing the S/L ratio (1:40 to 1:100) and increasing the duration (24–48 h) compared to previous SEPs^23^ did not completely dissolve the Fe (oxyhydr)oxides, especially Cr-goethite. Full dissolution was only achieved after further increasing the temperature to 75 °C. Time series experiments (Figure 2) revealed that all crystalline Fe (oxyhydr)oxides, except for Cr-goethite, were effectively dissolved within 2 h. Cr-goethite was only fully dissolved after 24 h, while chromite was unaffected even after 48 h. In comparison to prior dissolution of goethites using 6 M HCl at 25 °C,^29^ the optimized 6 M HCl treatment reduced the time to fully dissolve Al-substituted goethite from ∼220 to 2 h and increased the dissolution extent of Cr-goethite from <50% after 350 h to >98% after only 24 h.

Faster rates of dissolution were observed from pure minerals compared with their metal-substituted and natural counterparts. These findings are consistent with previous works on synthetic Fe (oxyhydr)oxides dissolved in HCl,^29,73,74^ where they attributed the slower dissolution rates of Cr- and Al-substituted Fe phases to the higher bond strength of the Me–OH/O bonds [e.g., Al(III)–O = 29.3 kJ mol^–1^; Cr(III)–O = 24.5 kJ mol^–1^] relative to Fe–OH/O [e.g., Fe(III)–O = 23.7 kJ mol^–1^].^75^ Moreover, very low rates of H_2_O exchange of Cr(III) has been suggested to explain the higher resistance of Cr-substituted goethite.^76^ In addition to metal substitution, larger particle sizes and smaller surface areas could explain the slower dissolution rates of natural samples.

We also tested the potential of the selected 24 h hot 6 M HCl treatment in extracting Fe from sheet silicates that could co-occur with the Fe (oxyhydr)oxides in the Ni laterites.^77,78^ Significant amounts of Fe (65–70%) were extracted from nontronite and serpentine. We compared these with the boiling 12 M HCl treatment previously used to extract sheet silicate Fe^41^ and documented that the optimized 6 M HCl released 2.5 times higher Fe, most likely due to the higher S/L ratio and longer extraction duration. Overall, this demonstrates the ability of the 6 M HCl treatment to dissolve highly crystalline Fe-bearing phases, such as metal-substituted (oxyhydr)oxides and sheet silicates, without affecting the residual fraction hosted in chromite.

### Enhanced Efficiency of the Optimized SEP

**Figure 3:**
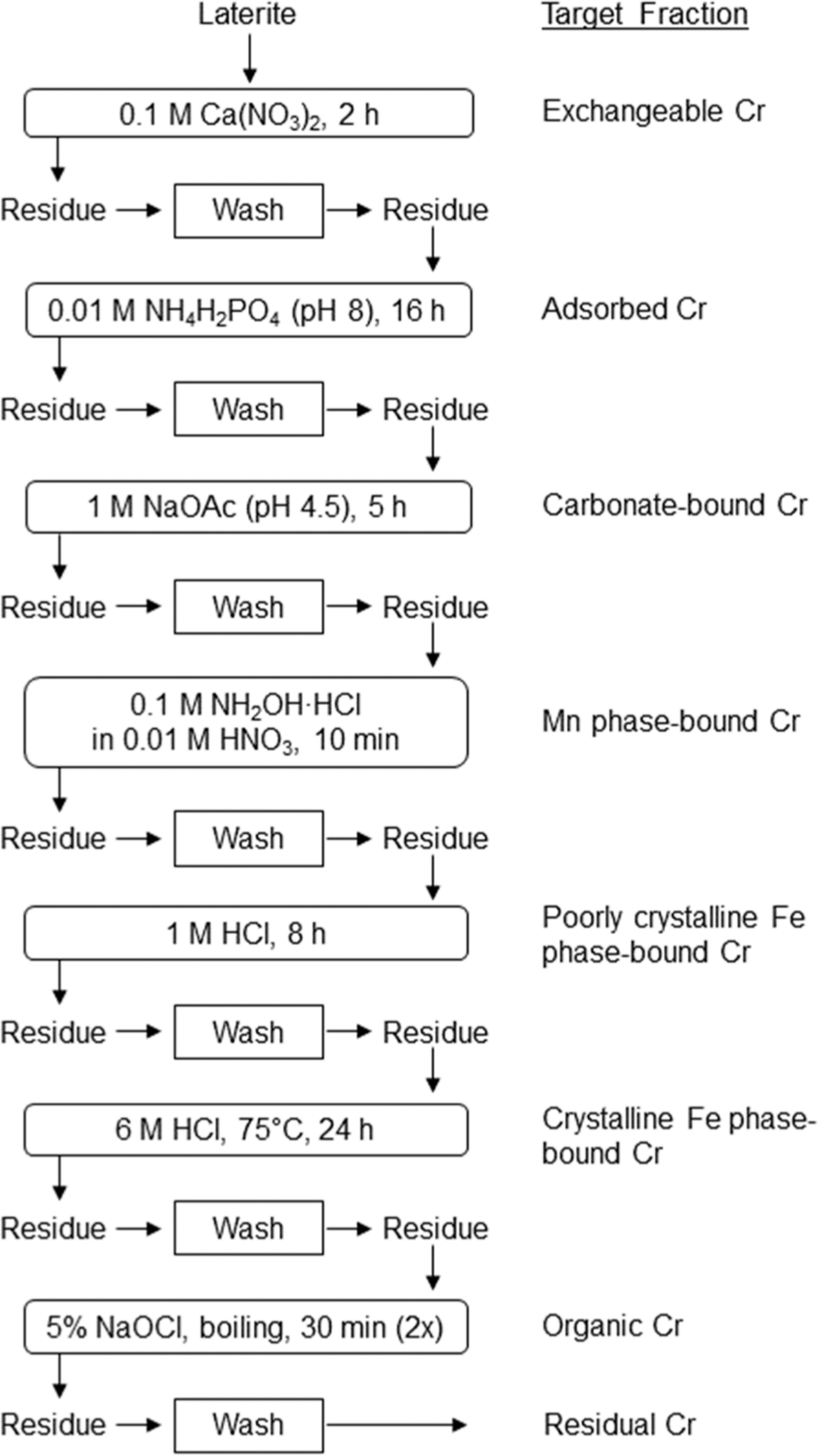
Optimized SEP for Cr in Fe-rich laterites. A solid-to-liquid ratio of 1:100 is applied for all except for organic Cr, where 1:20 was employed. Residual Cr is the difference between the total concentration and the sum of the preceding extractable fractions.

The resulting extraction scheme in Figure 3 was validated with mineral mixtures and nickel laterite CRMs (Table 1). Chromium recoveries from mixtures were mostly >89%, matching the results of the single extractions. Only the phosphate step targeting the smallest fraction of Cr showed a lower recovery. Moreover, sequential extraction of the CRMs showed well-targeted crystalline Fe phases (Figure S6).^24^

#### Easily Mobilizable to Non-Fe Phase-Bound Fractions

**Figure 4:**
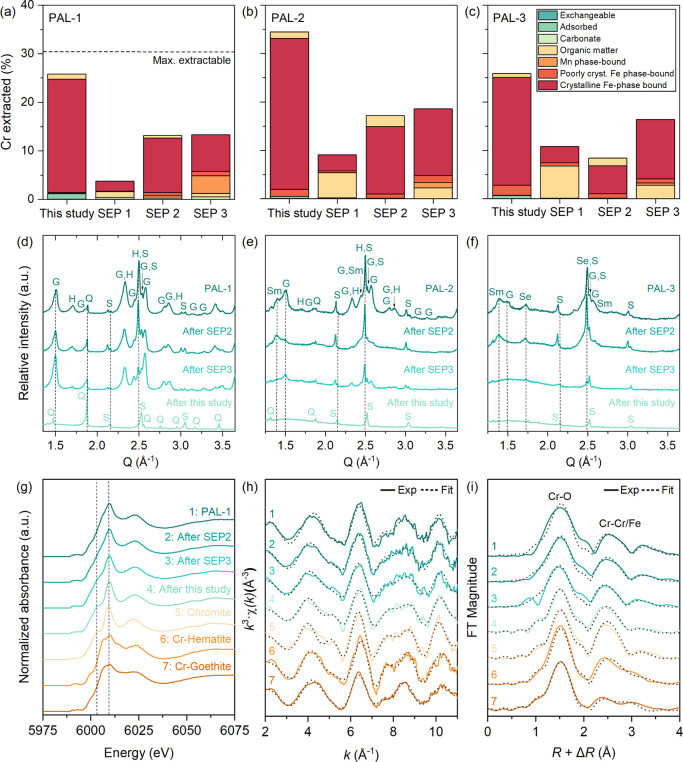
(a–c) Comparison of Cr partitioning (% of total concentration) and (d–f) residual fraction mineralogy of the Palawan Ni laterites (PAL-1—limonite, PAL-2—transition zone, PAL-3—saprolite) based on the optimized method (this study) and existing SEPs (SEP 1–3). The dashed line in (a) represents the maximum extractable Cr further discussed in Text S7. (g) Cr K-edge HERFD-XANES, (h) *k*^3^-weighted EXAFS spectra, and the corresponding (i) Fourier transforms (FT) of PAL-1 and its SEP residues and reference minerals. Vertical dashed lines in (g) denote features discussed in the text. Dotted lines superimposed on the solid lines in (h) and (i) denote shell-by-shell fits of the EXAFS data, respectively. Fit parameters are given in Table S6. G—goethite, H—hematite, S—spinel, Sm—smectite, Se—serpentine, and Q—quartz.

Applying the optimized scheme and three existing SEPs^23,28,32^ (Figure 4a–c) to the PAL laterites yielded very small amounts (<0.1% of total Cr) of exchangeable Cr. Our additional phosphate step leached up to 1% of the total Cr but with notable Fe extraction (up to 2.6%; Figure S7) in the transition and saprolite samples. During this step, we observed colloidal formation likely induced by the interaction of negatively charged phosphate ions and negatively charged surfaces of smectites identified through XRD (Figure S10). Some colloids might have passed through the filters, resulting in the detection of Fe. Another possible explanation is the dissolution of Fe minerals previously reported during phosphate extractions of arsenic, although the mechanism of dissolution remains unclear.^44,79^ In the case of the smectite-free limonite samples from Palawan, and from Zambales and Surigao (Figure S9), where up to 7% of total Cr was recovered in the phosphate step, no such Fe extraction was observed. Thus, care should be taken when interpreting phosphate extracted metals from samples containing clays.

In terms of organic Cr, the optimized SEP extracted comparable concentrations with SEP 2, which also used an oxidant (H_2_O_2_) after the crystalline Fe phase step but generally in lower amounts than SEP 3, where NaOCl was applied before Fe (oxyhydr)oxide dissolution. SEP 1 extracted the most (≤6.6%) due to the ligand-promoted dissolution of Cr-bearing, poorly crystalline Fe (oxyhydr)oxides by Na_4_P_2_O_7._^80−82^ This is supported by the anomalously high organic bound Fe (≤14%) (Figure S7) that correlates with the increasing trend of poorly crystalline Fe (oxyhydr)oxides down the laterite profile.^18^ All other SEPs only extracted <1% of total Fe in this step.

The optimized SEP and SEP 2 leached <1% of Cr using hydroxylamine hydrochloride, which targets the Mn phase-bound fraction. Conversely, SEP 3 extracted up to four times more Cr despite using the same reducing reagent. Unlike the two SEPs, the hydroxylamine hydrochloride treatment of SEP 3 is preceded by NaOCl. As previously discussed, NaOCl can oxidize mineral-bound Cr(III) to Cr(VI), promoting adsorption onto Mn (oxyhydr)oxides, which are known adsorbents of Cr(VI) species.^83^ This indicates that for Cr, applying an oxidant like NaOCl before the mineral dissolution step will not only overestimate organic Cr but also succeeding fraction(s) as a result of the carryover of oxidized Cr.

#### Fe Phase-Bound to Residual Fractions

Our SEP demonstrated the highest recoveries for Cr and Fe bound to Fe minerals. The 1 M HCl step dissolved increasing Cr (0.3–2%) and Fe (1–6%) fractions from PAL-1 to PAL-3, agreeing well with the increasing amount of poorly crystalline Fe (oxyhydr)oxides.^18^ SEP 1 showed the poorest recoveries for both poorly crystalline (<1% Cr, <1.5% Fe) and (<3.5% Cr, <12% Fe) crystalline fractions. Inefficient extraction by hydroxylamine hydrochloride^18^ and the nonselective Na_4_P_2_O_7_ step may account for these low recoveries. As discussed earlier, the latter can also retard the dissolution of Fe (oxyhydr)oxides.

Complete dissolution of Fe (oxyhydr)oxides by our optimized 6 M HCl step yielded up to an 11-fold increase in crystalline Fe phase-bound Cr and Fe relative to existing SEPs. Residues after this step (Figure 4d–f) reveal the absence of Fe (oxyhydr)oxides, showing only chromite and quartz signals in XRD and SEM (Figure S11). In the limonite residue where chromite is predominant, we have estimated the maximum extractable Cr (Text S7) and showed that our SEP yields the highest extraction efficiency, closest to the maximum value (Figure 4a) and shows ∼85% recovery. In contrast, residues from the two most efficient existing SEPs (2 and 3) show the incomplete dissolution of goethite and hematite, especially in the limonite sample. While our 6 M HCl step recovered 78% of total Fe from PAL-1, both the widely used citrate-bicarbonate-dithionite and 6 M HCl steps of SEPs 2 and 3, respectively, only recovered half.

Chromium K-edge XAS of the PAL-1 residues unveils how the different SEPs affect the dissolution of Cr. The Cr K-edge HERFD-XANES spectrum of the initial sample shows features common to chromite, Cr-hematite, and Cr-goethite, while our SEP’s residue displays features (dotted lines in Figure 4g) analogous only to chromite. Chromium EXAFS fitting of PAL-1 (Figure 4h,i, Table S6) revealed structural incorporation in chromite and Fe (oxyhydr)oxides and resulted in 7.5 neighboring oxygen (O) atoms at 2.00 Å, 4.8 Me_1_ at 3.05 Å, 6.0 Me_2_ at 3.27 Å, and 7.7 Me_3_ at 3.48 Å, where “Me” cations correspond to Cr and Fe. These cations have close atomic numbers and contribute similarly to the EXAFS signal,^84^ and therefore cannot be distinguished from each other. The Cr–O distance is consistent with the octahedral coordination of Cr(III) in chromite and Fe (oxyhydr)oxides but has a slightly higher coordination number (CN) that could be explained by the relatively high value of the correlated Debye–Waller factor (σ^2^) and uncertainty (20–25%) of EXAFS CNs.^85^ The 3.05 Å Cr–Me_1_ distance is similar but slightly longer than the second shell Cr–Cr/Fe distances of chromite and Cr–Fe (oxyhydr)oxides (2.97–2.98 Å). Rather, it is analogous to the average of the second shell distances and the Cr–Fe_E2_ distance of Cr-goethite (3.11 Å). Such strongly overlapping FT peaks often occur in natural heterogeneous samples and complicate the EXAFS fitting.^86^ Similarly, the Cr–Me_2_ distance is likely the average of 3.11 Å and the distances of corner-shared Cr–Fe atoms of Cr-goethite and Cr-hematite (3.41–3.44 Å), resulting in a single peak at 3.27 Å. These corner-shared distances of Fe (oxyhydr)oxides and that of chromite (Cr–Fe_c_ = 3.52 Å) could account for the fourth shell of PAL-1 at 3.48 Å. Only the outermost shell of Cr-hematite (∼3.7 Å) could not be fitted in PAL-1. This may be due to the heterogeneity of Cr location as observed in previous EXAFS fitting,^87^ where the outer shells of hematite were removed from the fit of a sample containing both hematite and goethite.

The PAL-1 residues after SEP 2 and 3 were similarly fitted with four shells, while the residue after our SEP was best fitted with only three shells. Cr–Me_1_ distances considerably differ and exhibit a decreasing trend from the initial value: SEP 2 (3.02 Å) > SEP 3 (2.99 Å) > optimized SEP (2.96 Å). This indicates decreasing (down to nonexistent) contributions from the Cr–Fe_E2_ shell of Cr-goethite and signifies the dissolution of Cr-bearing Fe (oxyhydr)oxides from the previous extraction steps. The Cr–Me_2_ shells of SEP 2 (3.23 Å) and SEP 3 (3.20 Å) residues also show a similar trend, while our SEP’s residue lacks this atomic correlation. Instead, our SEP’s residue has a second shell distance of 3.49 Å and an overall fit matching the local bonding environment of Cr in chromite^88,89^ only (Table S6). Such findings emphasize (1) the importance of laterite Fe (oxyhydr)oxides as hosts for Cr, and (2) previous SEPs only partially dissolve these minerals and underestimate their contributions.

Even in samples with smaller amounts of Fe minerals (PAL-2 and PAL-3), SEPs 2 and 3 exhibited limited dissolution of these phases (Figure 4e,f). On the other hand, our SEP completely dissolved these Fe phases, leaving chromite, quartz, and traces of amorphous silicate, showing a broad XRD peak at 1.57 Å. SEM-EDS confirms the presence of these Cr-free silicate phases (Figure S11b,c). With the optimization of Fe phase-bound extraction steps, our SEP significantly increased the recovery of extractable Cr, providing the best estimate of Cr in the Ni laterites. Additionally, it significantly increased the recovery of other equally important metals such as Mn and Ni (Figure S8).

Further tests on Zambales and Surigao limonites (Figure S9) revealed that our SEP yielded consistent high total recoveries for extractable and non-residual Cr (38–48%) and Fe (82–89%). In these samples, we highlight the importance of including the phosphate step and optimizing the crystalline Fe phase-bound step, especially because these steps extract the dominant pools for Cr. Adsorbed Cr comprised a maximum of 7% of the total Cr, while the crystalline Fe phase-bound fraction comprised the largest non-residual pool for Cr (29–44%) and Fe (82–89%).

### Environmental Implications

Existing SEPs applied to tropical soils fail to adequately partition Cr from Fe-rich laterites because they do not consider the different species of Cr and its ability to stabilize the crystal structure of Fe (oxyhydr)oxides. By robust calibration with appropriate mineral standards, we optimized and validated a SEP (Figure 3) that is reproducible, efficient, and selective for Cr in such Fe-rich materials. Identifying the binding sites of Cr is crucial for assessing its bioavailability, potential mobility, and transport mechanisms from laterites all the way to drinking and groundwaters. Our newly optimized SEP offers an important tool for monitoring and predicting pathways for Cr release that could ensue due to changes in environmental conditions (e.g., pH, redox, etc.). Our results also point to possible best practices for managing Cr-rich laterites, where the mobilization of Cr through weathering and mining may be linked to downstream containment and remediation efforts.

Exchangeable Cr targeted by Ca(NO_3_)_2_ represents easily mobilizable Cr in the presence of elevated salt inputs such as during saltwater interaction^90^ or irrigation.^91^ Adsorbed Cr could be liberated by phosphorus sources such as agricultural drainage.^90^ In a study by Becquer et al.,^16^ increased Cr concentrations in soil solutions was correlated to the desorption of Cr(VI) by phosphorus fertilizer inputs. Thus, accounting for adsorbed Cr is vital, especially in areas affected by agriculture and rehabilitation in the case of mining areas.

Chromium incorporated in Mn- and Fe-phases are more conservative pools and were only leached by reductive dissolution (NH_2_OH·HCl) and protonation (HCl). Thus, potential Cr release may occur under reducing (e.g., by bacterial activity)^91^ and acidic conditions (e.g., by organic acids). For example, common organic acids, such as oxalate and citrate, have been found to solubilize Cr-bearing goethite.^92^ Reducing and acidic conditions are also used in the hydrometallurgical processing of laterites (e.g., reductive bioleaching^93^ and high-pressure acid leaching^94^). In such cases, Mn and predominant Fe (oxyhydr)oxides are dissolved to solubilize associated Ni and Co, and knowledge of the extractable Cr from these phases is crucial in monitoring downstream processes and developing strategies for the immobilization of the leached Cr. Moreover, we demonstrated that interaction with strong oxidants such as NaOCl could potentially release Cr from Cr-bearing Fe (oxyhydr)oxides. NaOCl is extensively used in water treatment and has been found to oxidize Cr(III) to Cr(VI) during chlorination of drinking water.^67^ Our results warrant further research to assess the occurrence of Cr during drinking water treatment in lateritic areas. Furthermore, we were able to distinguish the extractable fractions from the residual chromite-bound Cr, which represents the weathering-resistant and most stable pool for Cr.

Using our optimized SEP, we demonstrated through the example of Philippine Ni laterites that these Fe-rich materials are significant sources of easily mobilizable and toxic Cr(VI) comprising up to 7% of total Cr. These fractions correspond to 30–1192 mg kg^–1^ Cr(VI) and are comparable to Cr(VI) detected in laterites from New Caledonia (≤358 mg kg^–1^)^19^ and Brazil (≤1014 mg kg^–1^).^30^ We also highlight the predominant association and structural incorporation of Cr as Cr(III) in Fe (oxyhydr)oxides, suggesting a potential release of Cr during the hydrometallurgical processing of laterites. The quantification of these important Cr reservoirs is crucial in ensuring the meticulous and sustainable management of laterite mining and processing regions.

## Supporting Information Available

Methods and mineral characterization; elemental composition of mineral standards; extraction protocols; dissolution efficiencies and Cr recoveries of mineral standards and laterites; EXAFS fitting results; and supporting XRD, FTIR, SEM data, and micrographs (PDF)
