# Supplementary material for: Partitioning and Mobility of Chromium in Iron-Rich Laterites from an Optimized Sequential Extraction Procedure
Source: Environ Sci Technol. 2024 Mar 29;58(14):6391–401. doi: 10.1021/acs.est.3c10774 (PMC11008241; doi:10.1021/acs.est.3c10774)
Supplement: Supplementary file 1 — es3c10774_si_001.pdf [file es3c10774_si_001.pdf]

Supporting information for

Environmental Science & Technology

Partitioning and mobility of chromium in iron-rich laterites from an optimized sequential extraction procedure

Ruth Esther G. Delina[1,2], Jeffrey Paulo H. Perez[1], Jessica A. Stammeier[1], Elena F. Bazarkina[3,4], Liane G. Benning[1,2]*

Affiliations:

[1] GFZ German Research Centre for Geosciences, Telegrafenberg, 14473 Potsdam, Germany

[2] Department of Earth Sciences, Freie Universität Berlin, 12249 Berlin, Germany

[3] The Rossendorf Beamline at ESRF, The European Synchrotron, CS 40220, 38043 Grenoble Cedex 9, France

[4] Institute of Resource Ecology, Helmholtz-Zentrum Dresden-Rossendorf, Bautzner Landstraβe 400, 01328 Dresden, Germany

*Corresponding author: rdelina@gfz-potsdam.de

This supporting information contains: 23 pages, 6 tables, and 11 figures.

Table of Contents

S1. Supporting materials and methods and mineral characterization……………………………………….S2

S2. Supplementary Tables……………………………………………………………………………………………….....S8

S3. Supplementary Figures……………………………………………………………………………………………….S13

References…….S21

S1. Supporting materials and methods and mineral characterization

Text S1. Characterization of Ni laterites

All samples were crushed and sieved to <63 µm prior to analyses. The mineralogy of the Ni laterite samples was confirmed through X-ray diffraction (XRD). Powdered samples were loaded inside 0.5-mm ID capillaries, and then analyzed in Debye-Scherrer geometry using a STOE STADI P diffractometer (Ag Kα radiation; λ = 0.5594 Å) equipped with a curved Ge (111) monochromator and two DECTRIS MYTHEN2 R detectors. XRD patterns were recorded over a *Q*-range of 0 to 13.42 $Å^{-1}$, with each sample measurement taking 2 h. Quantitative mineralogical analysis was performed through Rietveld refinement using the GSAS-II software.[1] Infrared (IR) spectroscopy of selected samples was also performed using a ThermoFisher Nicolet iS5 FTIR spectrometer with an iD7 diamond attenuated total reflectance accessory. The IR spectra were collected in the 4000-400 cm^{-1} range after coadding 64 scans collected at a resolution of 4 cm^{-1}. Element concentrations of the Zambales and Surigao Ni laterites were determined by inductively coupled plasma optical emission spectrometry (ICP-OES) using an Agilent 5110 spectrometer, following total digestion after standard Na_2O_2 fusion.[2] In brief, 0.1 g finely ground sample was fused with 0.6 g Na_2O_2 in a vitreous carbon crucible at 480°C. The sinter cake was dissolved in ultrapure water. Undissolved residues were separated by centrifugation, dissolved in HCl and added to the supernatant. Concentrations were determined using standard addition. Blanks were determined on a procedural blank and included in the calibration. The precision of the methodology was determined on CRM OREAS 182, 185 and 190.

Text S2. Synthesis and preparation of mineral standards

Metal-substituted Fe (oxyhydr)oxides were synthesized at mole fractions [X = Me/(Fe+Me), where Me = Al or Cr] up to 0.20, based on known compositions of Fe (oxyhydr)oxides in Fe-rich soils.[3,4]

Ferrihydrite. Metal-substituted 2-line ferrihydrites were prepared by dropwise addition (2.9 mL min^{-1}) of 1 M NaOH to 150 mL of mixed solution of 0.17 M $Fe(NO_3)_3·9H_2O$ and either 0.03 M $Al(NO_3)_3·9H_2O$ or $Cr(NO_3)_3·9H_2O$ until pH 7 was reached.[5] The initial pH of the mixed solutions are very acidic (pH 1.5).

Goethite. Al-goethite was made by quickly adding 100 mL of 0.2 M $Fe(NO_3)_3·9H_2O$ to a mixture of 120 mL of 0.06 M aluminate solution and 165 mL of 1 M NaOH. The aluminate solution was prepared by adding 500 mL of 0.1 M $Al(NO_3)_3·9H_2O$ and 300 mL of 1 M NaOH. The resulting suspension was aged at 70°C for 14 days.[5] Cr-goethite was synthesized similar to Al-goethite but with a longer aging time (120 days) and an alkaline Cr solution made by mixing 600 mL of 0.1 M $Cr(NO_3)_3$ solution with 360 mL of 1 M NaOH. It was prepared by adding 45 mL of 0.2 M $Fe(NO_3)_3·9H_2O$ to a mixed solution of 135 mL of the

alkaline Cr solution and 64 mL of 1 M NaOH.[5] Adsorbed ions and amorphous phases were removed from the Cr-goethite precipitates by a 2 h treatment with 3 M H_2SO_4 at 50°C and at a solid:liquid (S:L) ratio of 1:100. The washed precipitate was dried at 50°C for 24 h.[6]

Hematite. Al-substituted hematite was prepared by combining 160 mL of 0.1 M $Fe(NO_3)_3{\cdot}9H_2O$ and 40 mL of 0.1 M $Al(NO_3)_3{\cdot}9H_2O$ and adjusting the pH of the mixture (initial: pH 1.8) to 7 by dropwise addition of 1 M NaOH. The precipitate was washed three times and resuspended with Milli-Q water (~18.2 MΩ·cm) at pH 7 adjusted with NaOH. The suspension was stored at 80°C for 65 days.[5] Cr-hematite was formed by vigorously mixing 3.43 g of $Fe(NO_3)_3{\cdot}9H_2O$ and 0.6 g of $Cr(NO_3)_3{\cdot}9H_2O$ into a 0.01 M HNO_3 solution pre-heated at 98°C.[7] The slurry was stored for 25 days at 98°C and washed with 0.25 M HCl (1:100) for 2 h to remove adsorbed ions and amorphous materials. The Cr-hematite precipitates were washed with Milli-Q water until a pH >5 was reached and then oven dried at 70°C.[8]

Cr(VI)-adsorbed Fe (oxyhydr)oxides. Cr(VI)-adsorbed ferrihydrite, goethite, and hematite were prepared by reacting 500 mg of the minerals with 100 mg L^{-1} Cr(VI) solution (pH 7) in a 100 mL headspace vial shaken for 24 h at 150 rpm.

All other syntheses were performed in perfluoroalkoxy (PFA) reactors with a magnetic stirrer. After each experiment, the samples were transferred to polypropylene (PP) bottles or centrifuge tubes and washed with Milli-Q water to remove remaining electrolytes through four to eight cycles of centrifugation (10,052*g*, 10 min) until the total dissolved solids (TDS) of the supernatant is constantly low (~5 mg L^{-1}). Unless stated otherwise, all synthetic minerals except for ferrihydrite were dried in a vacuum desiccator. Poorly crystalline minerals such as ferrihydrite were freeze-dried to prevent possible transformation to crystalline Fe (oxyhydr)oxides like goethite and hematite.

The purity of the phases was examined through XRD and IR spectroscopy described above. The reference minerals were also observed under a FEI Quanta 3D FEG scanning electron microscope (SEM) coupled with an energy dispersive spectroscopy (EDS) system. Prior to analysis, the samples were mounted onto a double-sided carbon tape attached to SEM stubs and carbon-coated using a Leica EM ACE600 sputter coater. SEM images were collected at high vacuum mode, 20 kV and 60 pA using a Everhart Thornley secondary electron detector. Elemental composition of the minerals was determined using ICP-OES (Varian 720-ES) after aqua regia digestion. Note that not all added metals were incorporated in the Fe (oxyhydr)oxides during their formation as shown in the differences in intended and final mol. % substitution (**Table S1**).

Natural minerals. Magnetite from the mineral collection of the Institute of Applied Geosciences of Karlsruhe Institute of Technology, and chromite sample from the UG2 chromitite layer of the Bushveld Igneous Complex of South Africa were used in this study. Fe sheet-silicates such as nontronite (NAu-2) was procured from the Source Clays Repository of The Clay Minerals Society. The clay sample was fractioned to <2µm through a series of centrifugation adapted from Jackson[9] and washed with dilute HCl (0.25 M) for 2 h and rinsed seven times with Milli-Q water to remove potential impurities. The serpentine used in this study was obtained from the Berliner Mineralien Zentrum. Mineralogical and geochemical characterization of the samples were conducted using XRD and X-ray fluorescence (XRF), respectively. Fused beads were analyzed with a Malvern Panalytical AXIOS XRF. Reproducibility was determined on three certified reference materials (CRMs) and is within the analytical precision, which is better than 2% for main elements and better than 10% for trace elements.

Figure S1, **S2**, and **S3** show the XRD, IR, and SEM results, respectively, while **Table S1** summarizes the details and composition of all the mineral standards used in this study.

Text S3. Justification for the addition of the phosphate step

Most sequential extraction procedures used to study Cr partitioning in laterites have been originally designed for cationic metal species. However, Cr can exist as oxyanions of Cr(VI) (e.g., $HCrO_4^-$, CrO_4^{2-}), which under the common pH of Fe (oxyhydr)oxide-rich laterites (close to pH 5),[10] will be primarily controlled by adsorption/desorption reactions.[11] Phosphate extractions at pH 5 to 8[12-15] have been used for single extraction of Cr(VI)[12,16,17] to desorb Cr(VI) oxyanions by competitive adsorption.[18-20] Previous works have shown maximum sorption of chromate onto Fe (oxyhydr)oxides at pH close to 5,[21] leading to chromate re-adsorption when phosphate extraction was done at pH 5.[16] X-ray absorption near edge structure (XANES) measurements by Fandeur et al.[16] revealed that only 50% of Cr(VI) from a lateritic regolith was recovered using a phosphate solution at pH 5. Hence, we employed an alkaline (pH 8) 0.01 M $NH_4H_2PO_4$ treatment for 16 h.[22,23] At this pH, adsorption of Cr(VI) onto Fe (oxyhydr)oxides was found to be minimum.[21] Additionally, comparative studies on phosphate extractions applied at pH 7 and 8 revealed a higher desorption capacity from the higher pH solution.[15] Furthermore, phosphate extractions have been employed at concentrations ranging from 0.01 to 0.1 M.[15,22,24] Here, we used a lower concentration wherein minimum Fe (oxyhydr)oxide alteration is expected and extraction equilibrium has been achieved at 16 h, as reported in a previous study.[22] The addition of the phosphate step can distinguish the easily mobilizable Cr(VI) from the exchangeable Cr which represents weakly-sorbed cations (e.g., Cr(III)) that can be released from mineral surfaces (e.g., clays) through ion-exchange processes.

Text S4. Single extractions

Extractions were performed at solid-to-liquid (S:L) ratios from 1:5 to 1:100 using a temperature-controlled orbital shaker (250 rpm) or manual shaking when in a boiling water bath. For example, 50 mg of the reference minerals, equivalent to 50% of a single phase in a 100 mg sample was mixed with 2 to 10 mL of the extractants. The resulting solutions were centrifuged at 10,052*g* for 10 min and filtered using 0.2-μm polyvinylidene fluoride (PVDF) syringe filters. For time-sensitive (e.g., 5 min) extractions, vacuum filtration using 0.2-μm polycarbonate membrane filters were directly conducted after extraction. All, except for the HCl extracts, were acidified with HCl (Aristar® VWR) and all samples were stored at 4°C prior to analysis. Elemental concentrations of the solutions were determined via ICP-OES (Varian 720-ES) following the method described by Perez et al.[25] and detailed below.

Text S5. Inductively coupled optical emission spectrometry (ICP-OES)

Samples analyzed using the Varian 720-ES ICP-OES system were prepared by gravimetric dilution using 0.3 M HNO_3 containing 1 mg g^{-1} Cs acting as an ionization buffer and an internal standard. Indium (1.33 μg g^{-1}) was used as an internal standard for the sequential extraction supernatants while Sc (1 μg g^{-1}) was used for the aqua regia digested samples and single extraction supernatants. Calibration standards were prepared using multi-element and single element standards (e.g., Merck Certipur®, Sigma Aldrich, etc.) mixed with 1 mg g^{-1} Cs, 0.3 M HNO_3, and appropriate amounts of HCl for sample matrix matching. For each analysis, Ar, Cs, and In or Sc were monitored for instrument stability and drift. Instrumental statistical LoD (3SD above background) or limits of quantification (LoQ = 10SD) were quantified using ≥5 repeat analysis of the sample diluent (0.3 M HNO_3 + 1 mg g^{-1} Cs + 1.33 μg g^{-1} In or 1 μg g^{-1} Sc). Analytical uncertainties of analyte concentrations were determined by ≥5 repeat analysis of a QC solution.

Text S6. High-energy resolution fluorescence detected X-ray absorption spectroscopy (HERFD-XAS)

Cr K-edge X-ray absorption near edge structure (XANES) and extended X-ray absorption fine structure (EXAFS) spectra were collected on the bending magnet ROBL BM20 beamline of the European Synchrotron Radiation Facility (Grenoble, France).[26] The storage ring was operated at 6 GeV with a ~200 mA current in 7/8+1 filling mode. The energy of the incoming beam was selected using a double Si(111) crystal monochromator with higher harmonics suppressed by two Si mirrors operating in the total reflection mode while the beam size was 50 × 2000 μm^2. Energy calibration was done using a Cr metal foil, setting the maximum of the first derivative of the main edge to 5989 eV. Samples and reference compounds were pressed in pellets, placed vertically, rotated 45° to the incident beam and analyzed under ambient conditions. Pressed pellets (7-mm diameter) were prepared by loading ~20 mg of the

powder sample in a Specac® mini-pellet press and sealing with a Kapton® polyimide tape. Depending on their stability, the samples were diluted with up to 5 mg of boron nitride (BN), resulting in a mixture of ~20 mg.

Due to the relatively low concentration of Cr, background fluorescence from Fe, and complex matrix of natural samples, the spectra were recorded in HERFD mode. The details about HERFD measurements and advantages for the environmental sciences were discussed previously.[27] In this study, a Johann-type X-ray emission spectrometer (XES) in a vertical Rowland geometry available at BM20[28] was equipped with spherically bent crystal analyzers with a 1 m bending radius, and a silicon drift X-ray detector (©Ketek). For our measurements, five Ge(211) crystal analyzers were aligned at the maximum of the Cr $K\alpha_1$ emission line (5414.9 eV) using (422) reflection and the 82.5° Bragg angle. To optimize the XES, the maximum of the corresponding non-resonant emission line of the Cr metal foil was selected. A helium-filled bag was placed to fill the optical path sample-crystal analyzers-detector to minimize the absorption of the fluorescence signal by air. A total experimental energy resolution of ~1.2 eV was estimated using the width of the elastic scatter peak at 5415 eV.

All HERFD-XAS spectra were normalized to the intensity of the incident beam measured using a Canberra photodiode and Kapton foil placed at the incident beam optical path before the sample. Depending on data quality, an average of 3 scans were performed on each sample, with an acquisition time of ~35 min/scan. Spectra were aligned, averaged, and background-subtracted using the ATHENA[29] and SIXpack[30] software.

Shell-by-shell fits (**Table S6**) were performed from 1.2 to 3.8 Å R+ΔR-space in SIXpack[30] using the algorithms derived from IFEFFIT.[31] The fitted Fourier transforms were filtered from the k^3-weighted EXAFS data by a Kaiser-Bessel window. Theoretical phase and amplitude functions for single and multiple scattering paths (Cr-O, Cr-O-O, Cr-Cr, Cr-Fe) were calculated using FEFF6[32] from the crystal structure of chromite.[33] Similar to previous work,[34] the passive electron reduction parameter, S_0^2, was constrained to a value of 0.7. The reference minerals (chromite, Cr-hematite, Cr-goethite) were chosen based on the dominant mineralogy of the limonite sample. The best fits of their EXAFS spectra were obtained by defining shells of neighboring atoms and then iterating the coordination numbers (CN), distances (R), and the mean squared atomic displacement parameter or Debye-Waller factors (σ^2). To reduce the number of degrees of freedom and fit-derived standard errors from highly correlated parameters (e.g., CN and σ^2), the CN of the references were fixed according to theoretical values. This fitting technique was used by previous studies[35-38] and results showed consistent local bonding environment of structurally incorporated elements in chromite,[39,40] hematite,[35,36] and goethite.[37] Only

the outer Cr-Fe shell of chromite was best fitted with a lower CN (i.e. 4) than literature value (i.e. 6)[39] probably due to multiple metal-substitution (e.g., Mg, Mn) in the natural specimen. Fitting of the limonite and SEP residues were performed based on their mineralogical data and single scattering paths of the references. Because of the heterogeneity of the samples, the coordination numbers were not constrained. Instead, the σ^2 was set to the value reported for the corresponding reference mineral scattering path to avoid high fit-derived standard errors in these fitting parameters. Fit quality was evaluated based on the R-factor: $R = \sum_i(data_i - fit_i)^2/\sum_i(data_i)^2$, where a value of <0.05 signifies a reasonable fit.[41]

Text S7. Estimation of maximum extractable Cr in limonite

To account for metal-substitution and natural heterogeneity of chromites, we determined the concentration of Cr in the residues through SEM-EDS. We collected single EDS spectrum from representative (i.e. size, morphology) chromite grains (n=15). The statistics of the composition of the grains in the limonite sample, PAL-1, are as follows:

	Concentration (wt.%)						
	Al	Cr	Fe	Mg	Mn	Si	Ti
Minimum	2.2	18.1	5.3	1.0	0.2	0.1	0.0
Maximum	10.5	48.6	16.3	5.4	1.6	15.5	0.8
Median	5.4	27.0	9.1	3.4	0.4	0.4	0.1
Average	5.3	30.0	10.5	3.5	0.6	1.8	0.2

The amount of maximum extractable Cr in the residue (i.e. chromite) was calculated as follows:

$$Maximum\ extractable\ Cr = \left(1 - \frac{C_{SEM}\ x\ Wt_{Res}}{C_{Tot}\ x\ W_{Tot}}\right) x\ 100$$

where,
C_{SEM} = median Cr concentration measured using SEM-EDS
Wt_{Res} = weight of residue
C_{Tot} = total Cr concentration of sample
Wt_{Tot} = total weight of sample

It is noteworthy that this quantification approach can only be applied to the limonite samples where the residue is mainly composed of chromite.

S2. Supplementary Tables

Table S1. Chemical composition of metal-substituted Fe (oxyhydr)oxides. Initial mol % substitution [Al or Cr/(Fe + Al or Cr)] was calculated from the synthesis procedure while the concentration and final mol % substitution were determined through ICP-OES.

Mineral references	Concentration (wt.%)			Initial mol % subs.	Final mol % subs.	Synthesis method
	Al	Cr	Fe			
Synthetic (pure)						
Ferrihydrite	-	-	56.6 (1.2)			5
Goethite	-	-	62.5 (2.0)			5
Hematite	-	-	61.9 (2.0)			42
Magnetite	-	-	69.1 (2.2)			5
Synthetic (substituted)						
Al-Ferrihydrite	4.0 (0.04)	-	51.1 (1.1)	15	14.0 (0.3)	5
Al-Goethite	2.2 (0.09)	-	55.8 (1.8)	27*	7.6 (0.4)	5
Al-Hematite	6.0 (0.06)	-	54.5 (1.2)	20	18.5 (0.4)	5
Cr-Ferrihydrite	-	7.9 (0.17)	47.7 (1.0)	15	15.0 (0.5)	5
Cr-Goethite	-	7.3 (0.21)	50.1 (1.6)	27*	13.5 (0.6)	5,6
Cr-Hematite	-	0.63 (0.01)	67.3 (1.4)	15	1.0 (0.03)	7,8
Synthetic (adsorbed)						
Cr(VI)-ads. Ferrihydrite	-	1.3 (0.02)	54.0 (0.7)			
Cr(VI)-ads. Goethite	-	0.31 (0.01)	59.5 (0.8)			
Cr(VI)-ads. Hematite	-	0.32 (0.01)	65.1 (0.8)			
Natural				Source		
Magnetite	0.09 (0.001)	-	58.5 (1.3)	Institute of Applied Geosciences of Karlsruhe Institute of Technology		
Chromite	14.4	33.0	18.4	UG2 chromitite layer, Bushveld Igneous Complex		
Nontronite	5.5	0.01	20.9	The Clay Minerals Society		
Serpentine	0.7	0.06	1.3	Berliner Mineralien Zentrum		

*reported Al/Cr for Fe substitution is <12 mol.%[5]

(#) – analytical uncertainty (<5% relative) based on multiple measurements ($n \geq 5$) of QC solutions.

Table S2. Reagents and operating conditions of existing SEPs applied to extractable, non-residual fractions. Each SEP is applied to 1 g of sample.

Step	Target Fraction*	Extractant	Conditions
SEP 1[43]**			
1	Exchangeable	30 mL 0.1 M $NaNO_3$	1.5 h, 25 °C
2	Carbonate	30 mL 1 M NaOAc (pH 5)	1.5 h, 25 °C
3	Organic matter	30 mL 0.1 M $Na_4P_2O_7$	1.5 h, 25 °C
4	Poorly crystalline Fe (oxyhydr)oxides	30 mL 0.25 M $NH_2OH \cdot HCl$ in 0.05 M HCl	1.5 h, 60 °C
5	Crystalline Fe (oxyhydr)oxides	30 mL 1 M $NH_2OH \cdot HCl$ in 25% CH_3COOH	1.5 h, 90 °C
SEP 2[44]			
1	Exchangeable	10 mL 0.1 M KCl	2 h, 20°C
2	Carbonate	10 mL NaOAc (pH 5)	5 h, 20°C
3	Mn (oxyhydr)oxides	10 mL 0.1 M $NH_2OH \cdot HCl$ (pH 2)	30 min, 20 °C
4	Poorly crystalline Fe (oxyhydr)oxides	10 mL 0.2 M $(NH_4)_2C_2O_4 \cdot H_2O$ + 0.2 M $H_2C_2O_4$ (pH 3)	4 h, 20 °C, dark
5	Crystalline Fe (oxyhydr)oxides	50 mL CB: $Na_3C_6H_5O_7$, $2H_2O$ (78.4 g/L) + $NaHCO_3$ (9.82 g/L); 1 g $Na_2S_2O_4$ (pH 7)	15 min in CB, 30 min after + $Na_2S_2O_4$; 80 °C
6	Organic matter	1) 3 mL 0.2 M HNO_3 - 8 mL 35% H_2O_2<br>2) 5 mL 3.2 M NH_4OAc (20% v/v HNO_3)	1) 5 h, 85 °C<br>2) 30 min, 85 °C
SEP 3[45]			
1	Exchangeable	15 mL 0.1 M $CaCl_2$	2 h, room temp
2	Adsorbed	30 mL 1 M NaOAc (pH 5)	5 h, room temp
3	Organic matter	5 mL NaOCl (pH 8.5)	30 min, 90-95 °C
4	Mn (oxyhydr)oxides	30 mL 0.05 M $NH_2OH \cdot HCl$ (pH 2)	30 min, room temp
5	Poorly crystalline Fe (oxyhydr)oxides	30 mL 0.2 M $(NH_4)_2C_2O_4 \cdot H_2O$ + 0.2 M $H_2C_2O_4$ (pH 3)	2 h, dark
6	Crystalline Fe (oxyhydr)oxides	40 mL 6 M HCl	24 h, room temp

*For clarity, names of target fractions were made consistent based on the nature of extractants. Please refer to respective sources for original terminologies.

**Each step except is repeated twice

Table S3. Fe and Al dissolution efficiencies of tested single extractions on Cr- and Al-bearing references. ads – adsorbed, FHY – ferrihydrite, Goe – goethite, Hem – hematite, Ox – (oxyhydr)oxides.

Extractant	Cr(VI)-ads FHY	Cr(VI)-ads Goe	Cr(VI)-ads Hem	Cr-FHY	Cr-Goe	Cr-Hem	Chromite
	Adsorbed Cr			Poorly cryst. Fe Ox	Crystalline Fe Ox		Residual
	Fe dissolution efficiency (%)						
0.1 M $Ca(NO_3)_2$	*bdl*	*bdl*	*bdl*	ND	ND	ND	ND
0.01 M $NH_4H_2PO_4$	*bdl*	*bdl*	*bdl*	ND	ND	ND	ND
5% NaOCl (1:5)	ND	ND	ND	*bdl*	*bdl*	*bdl*	*bdl*
5% NaOCl (1:20, 2x)	ND	ND	ND	*bdl*	*bdl*	*bdl*	*bdl*
0.5 M HCl, 4 h	ND	ND	ND	79.8 (1.9)	ND	ND	ND
1 M HCl, 4 h	ND	ND	ND	95.4 (2.2)	ND	ND	ND
1 M HCl, 8 h	ND	ND	ND	97.6 (2.3)	0.22 (0.01)	2.06 (0.05)	*bdl*
6 M HCl, 50°C, 48 h	ND	ND	ND	ND	51.8 (1.9)	98.0 (3.5)	*bdl*
6 M HCl, 75°C, 24 h	ND	ND	ND	ND	100 (4)	102 (4)	*bdl*

Extractant	Al-FHY	Al-Goe	Al-Hem	Al-FHY	Al-Goe	Al-Hem
	Poorly cryst. Fe Ox	Crystalline Fe Ox		Poorly cryst. Fe Ox	Crystalline Fe Ox	
	Fe dissolution efficiency (%)			*Al dissolution efficiency (%)*		
0.5 M HCl, 4 h	95.8 (2.2)	ND	ND	97.1 (1.2)	ND	ND
1 M HCl, 4 h	98.8 (2.3)	ND	ND	100 (1)	ND	ND
1 M HCl, 8 h	99.3 (2.3)	0.05 (2E-3)	5.61 (0.15)	99.5 (1.3)	0.57 (0.02)	9.13 (0.15)
6 M HCl, 50°C, 48 h	ND	103 (4)	103 (4)	ND	104 (5)	103 (5)
6 M HCl, 75°C, 24 h	ND	100 (4)	101 (4)	ND	100 (4)	100 (4)
5% NaOCl (1:5)	*bdl*	*bdl*	*bdl*	*bdl*	*bdl*	*bdl*
5% NaOCl (1:20, 2x)	*bdl*	*bdl*	*bdl*	*bdl*	*bdl*	*bdl*

Dissolution efficiency = (wt.% extracted ⁄ wt.% total) x 100
(#) – analytical uncertainty (<5% relative) based on multiple measurements ($n \geq 5$) of QC solutions
bdl – below detection limit; ND – no data

Table S4. A comparison of Fe extracted (%) after HCl extraction without and with prior PO_4 treatment. For the comparison, extractions were performed at the same duration. syn – synthetic, nat – natural.

Reference mineral	HCl extraction	HCl extraction after PO_4 treatment
Ferrihydrite	99 (2)	96 (3)
Goethite	104 (4)	69 (2)
Hematite	105 (5)	102 (4)
Magnetite (syn)	99 (4)	99 (1)
Magnetite (nat)	98 (4)	87 (2)
Chromite	*bdl*	*bdl*

(#) – analytical uncertainty (<5% relative) based on multiple measurements ($n \geq 5$) of QC solutions
bdl – below detection limit

Table S5. Chromium recovery from mixture of mineral standards after applying selected SEP steps.

Extractant	Mixture composition	Actual individual mineral wt.% Cr	Expected fraction wt.% Cr	Measured fraction wt.% Cr*
	Mixture 1			
Step 1: 0.1 M $Ca(NO_3)_2$				0.003
Step 2: 0.01 M $NH_4H_2PO_4$	Cr(VI)-ads Goe	0.02	0.03	0.02
	Cr(VI)-ads Hem	0.02		
Step 5: 1 M HCl, 8 h				0.06
Step 6: 6 M HCl, 75C, 24h	Cr-Goe	2.90	3.15	2.90
	Cr-Hem	0.25		
Residual	Chromite	3.34	3.34	3.54
	Mixture 2			
Step 1: 0.1 M $Ca(NO_3)_2$				
Step 2: 0.01 M $NH_4H_2PO_4$				
Step 5: 1 M HCl, 8 h	Cr-FHY	0.80	0.80	0.82
Step 6: 6 M HCl, 75C, 24h	Cr-Goe	2.89	2.93	2.61
	Cr-Hem	0.03		
Residual	Chromite	3.31	3.31	3.61
	Silica glass**			
	Mixture 3			
Step 1: 0.1 M $Ca(NO_3)_2$				
Step 2: 0.01 M $NH_4H_2PO_4$				
Step 5: 1 M HCl, 8 h	Cr-FHY	1.20	1.20	1.08
Step 6: 6 M HCl, 75C, 24h	Cr-Goe	0.74	0.74	0.80
Residual	Chromite	4.92	4.92	4.98
	Silica glass			

*Residual wt.% was represented by the (wt.% total Cr - ∑wt.% non-residual) ∕ wt.% Cr in chromite
**Silica glass spheres (high purity, Sigma-Aldrich) was added to Mixture 2 and 3 to mimic lower Fe containing samples

Table S6. Cr K-edge EXAFS fitting results summarizing the local coordination environment around a central Cr atom for the limonite sample (PAL-1) and residues (after SEP 2, SEP 3 and the optimized SEP in this study), and reference minerals.

Sample	Path	CN	R (Å)	σ^2 (Å^2)	ΔE_0 (eV)	*R*
PAL-1	Cr-O	7.5 (1.6)	2.00 (1)	0.006 (2)	-1.2 ± 2.4	0.0432
	Cr-Cr	4.8 (1.7)	3.05 (2)	*0.006*		
	Cr-Fe	6.0 (3.4)	3.27 (3)	*0.006*		
	Cr-Fe	7.7 (3.8)	3.48 (3)	*0.01*		
After SEP 2	Cr-O	6.7 (1.3)	1.99 (1)	0.005 (2)	-4.3 ± 2.2	0.0222
	Cr-Cr	4.6 (1.3)	3.02 (2)	*0.006*		
	Cr-Fe	3.6 (2.3)	3.23 (3)	*0.006*		
	Cr-Fe	4.7 (2.9)	3.46 (4)	*0.01*		
After SEP 3	Cr-O	6.7 (1.3)	1.98 (1)	0.006 (2)	-6.5 ± 2.4	0.0214
	Cr-Cr	4.4 (1.2)	2.99 (2)	*0.006*		
	Cr-Fe	3.3 (2.1)	3.20 (2)	*0.006*		
	Cr-Fe	4.4 (2.6)	3.43 (3)	*0.01*		
After this study	Cr-O	5.1 (0.8)	1.98 (08)	0.003 (1)	-7.0 ± 1.7	0.0201
	Cr-Cr	3.6 (1.2)	2.96 (1)	0.006 (2)		
	Cr-Fe	2.8 (1.4)	3.49 (4)	*0.01*		
Chromite	Cr-O	5.6 (1.3)	1.99 (1)	0.003 (2)	-6.2 ± 2.3	0.0497
	Cr-Cr_E	*6*	2.98 (2)	0.007 (1)		
	Cr-Fe_C	*4*	3.52 (5)	0.01 (05)		
Cr-Hematite	Cr-O	*6*	1.98 (1)	0.003 (1)	-3.3 ± 2.3	0.0280
	Cr-Fe_{F+E}	*4*	2.97 (2)	0.008 (1)		
	Cr-Fe_{C1}	*3*	3.44 (6)	0.01 (06)		
	Cr-Fe_{C2}	*6*	3.74 (8)	0.02 (1)		
Cr-Goethite	Cr-O	*6*	1.98 (09)	0.005 (05)	-3.6 ± 1.7	0.0204
	Cr-Fe_{E1}	*2*	2.97 (2)	0.006 (4)		
	Cr-Fe_{E2}	*2*	3.11 (3)	*0.006*		
	Cr-Fe_C	*4*	3.41 (6)	0.02 (1)		

Note: CN - coordination number, R - interatomic distance, σ^2 - mean-squared atomic displacement, ΔE_0 - change in threshold energy, and *R* - "goodness of fit" factor. Subscripts refer to: E – edge-shared, F – face-shared, C – corner-shared. Numbers in parenthesis are fit-determined standard errors on the last decimal place except when the error has the same significant figures as the fitted value. Constrained parameters appear in italics.

S3. Supplementary Figures

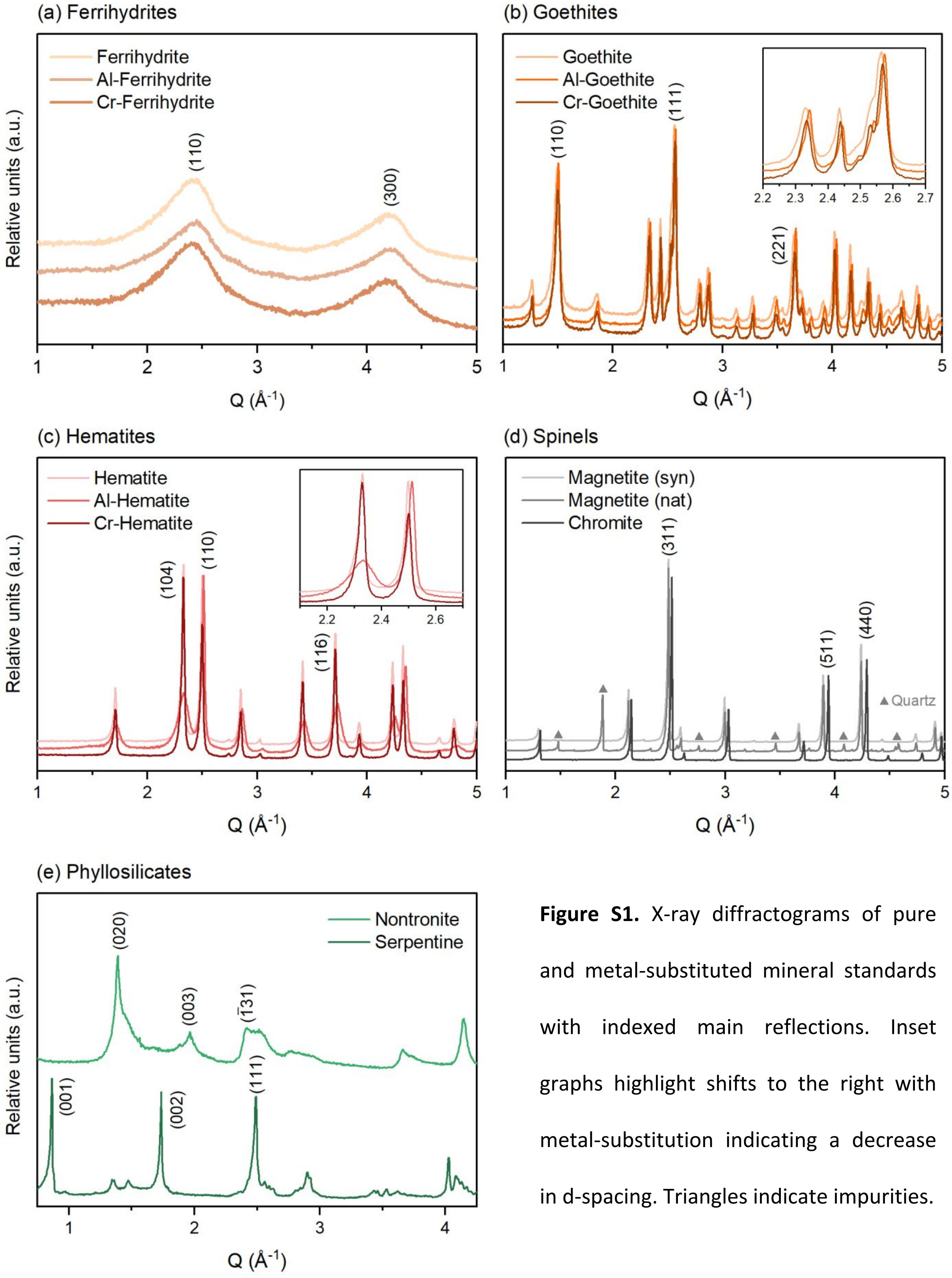

Figure S1. X-ray diffractograms of pure and metal-substituted mineral standards with indexed main reflections. Inset graphs highlight shifts to the right with metal-substitution indicating a decrease in d-spacing. Triangles indicate impurities.

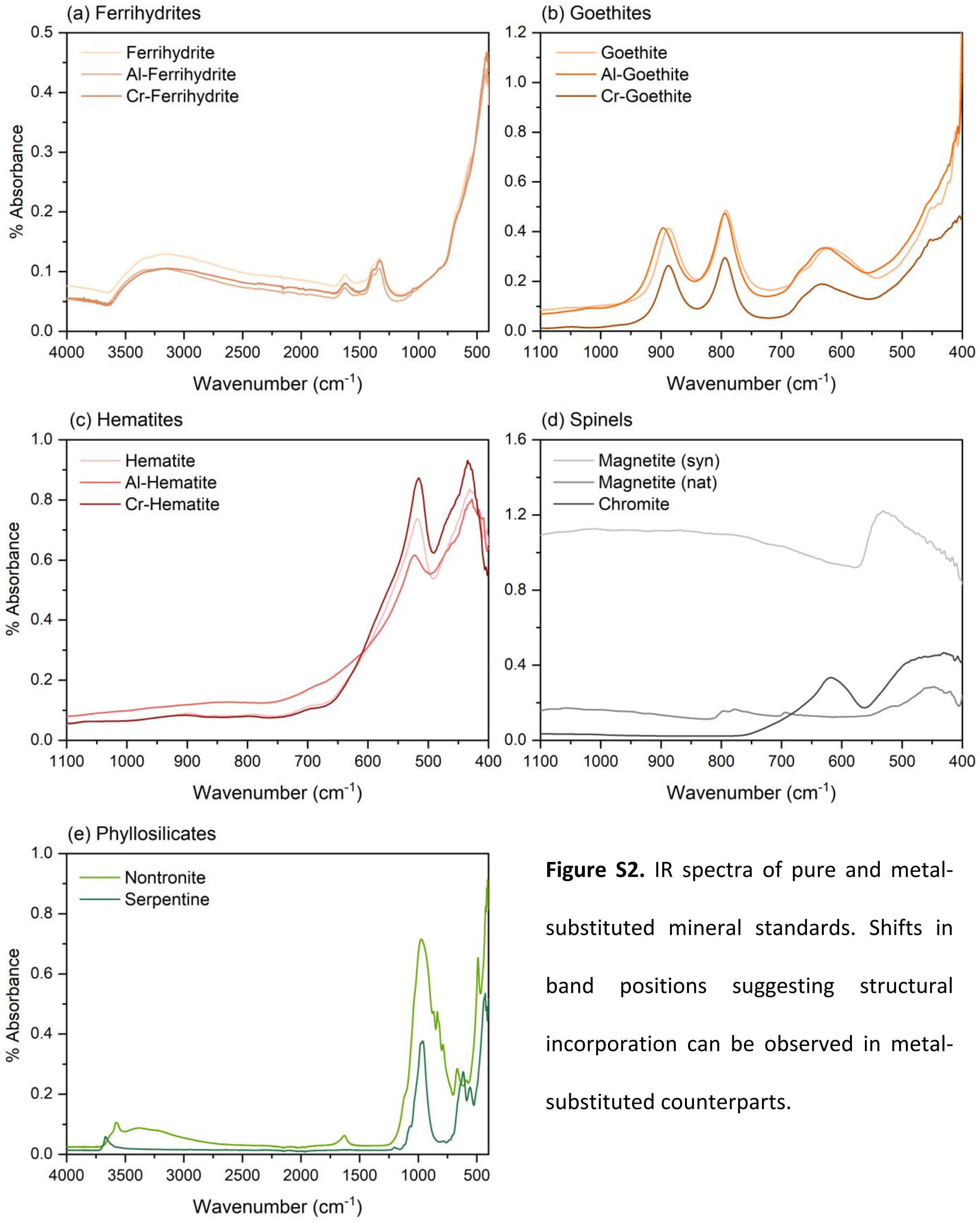

Figure S2. IR spectra of pure and metal-substituted mineral standards. Shifts in band positions suggesting structural incorporation can be observed in metal-substituted counterparts.

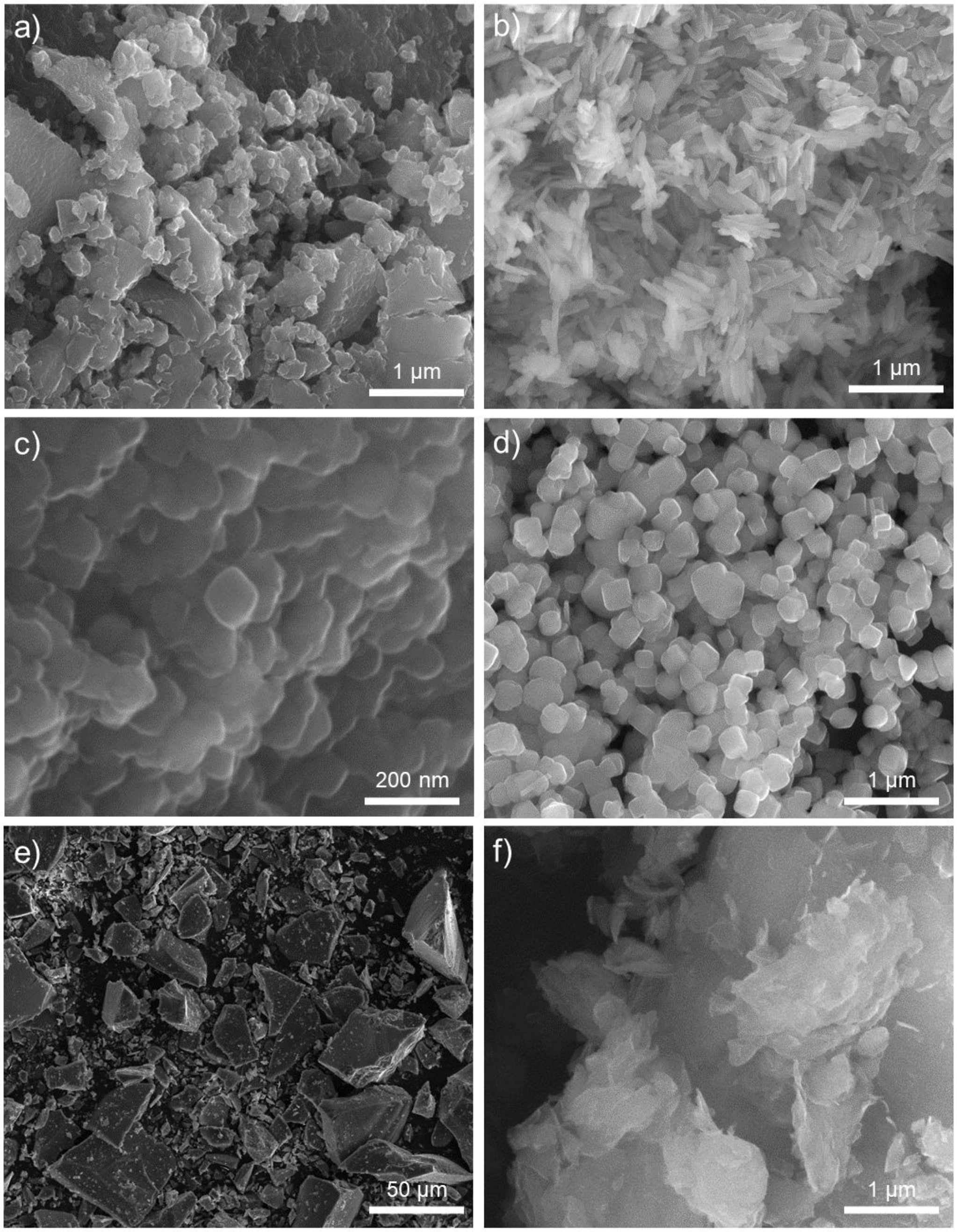

Figure S3. SEM images of representative samples: (a) ferrihydrite, (b) acicular goethite, (c) rhombus-like shaped hematite, (d) cubic magnetite, (e) chromite grains, and (f) nontronite clay.

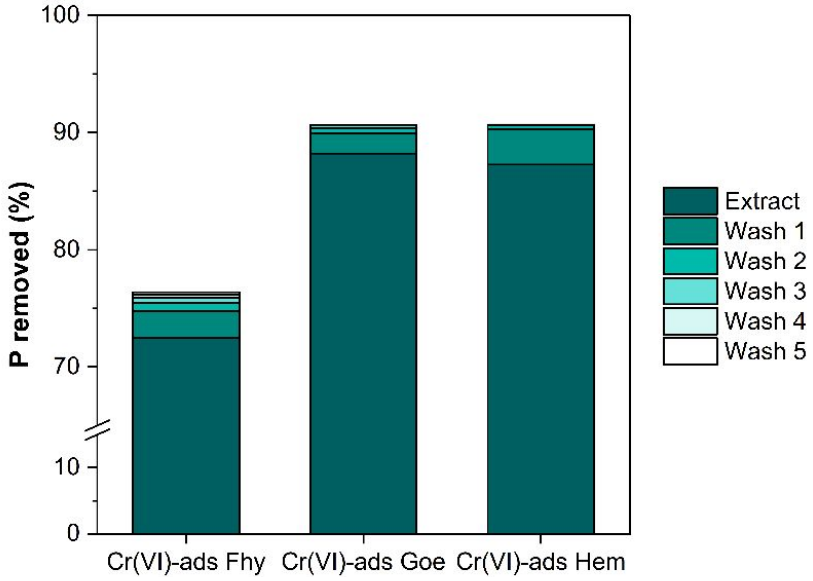

Figure S4. Phosphorus removed from Cr(VI)-adsorbed minerals after serial H_2O washing. Fhy – Ferrihydrite, Goe – Goethite, Hem – Hematite.

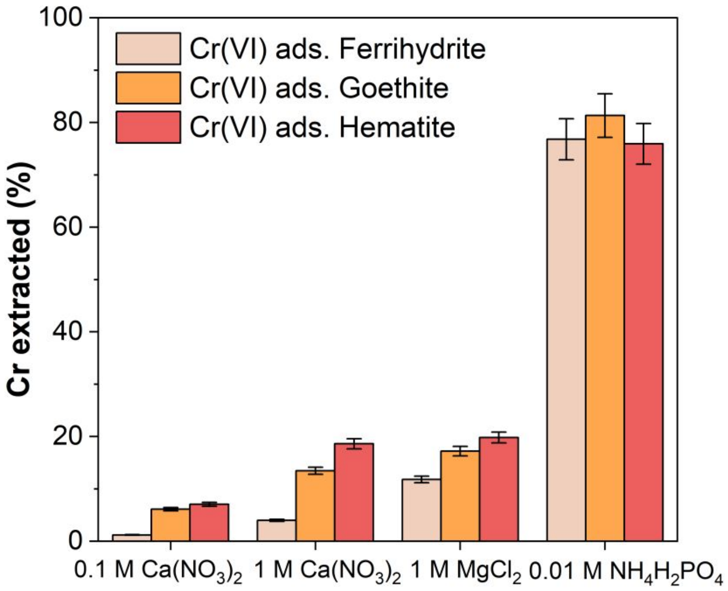

Figure S5. Chromium extracted from Cr(VI)-adsorbed Fe (oxyhydr)oxides using different reagents. Error bars indicate analytical uncertainty (<5% relative) based on multiple measurements (n = 5) of QC solutions.

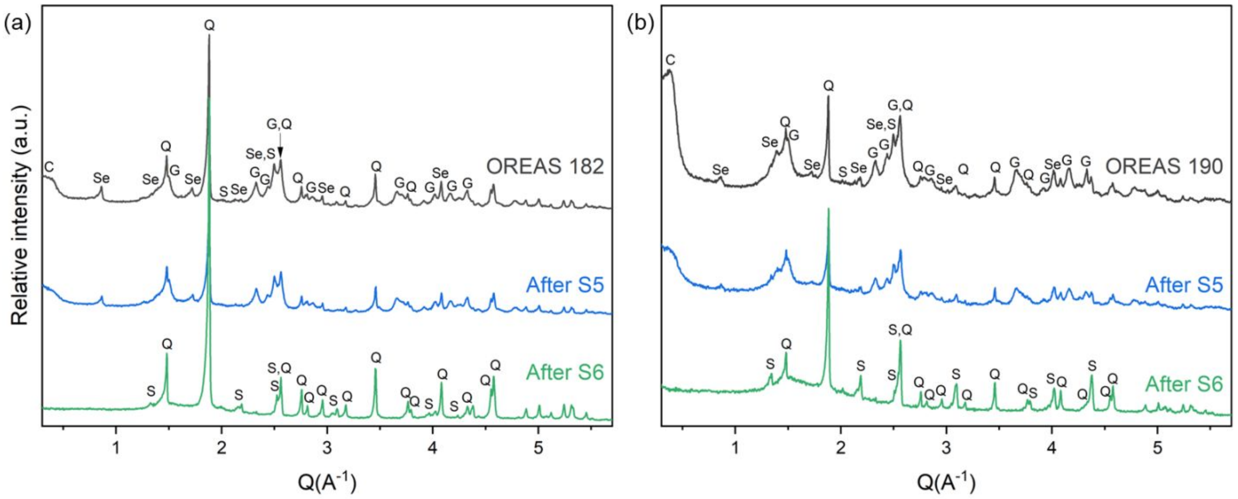

Figure S6. Comparison of the residues of the laterite CRMs (a) OREAS 182 and (b) OREAS 190 after Step 5 (poorly crystalline Fe phase-bound) and Step 6 (crystalline Fe phase-bound) of the optimized SEP showing the complete dissolution of goethite (G), serpentine (Se), and clays (C) leaving quartz (Q) and spinels (S) after Step 6.

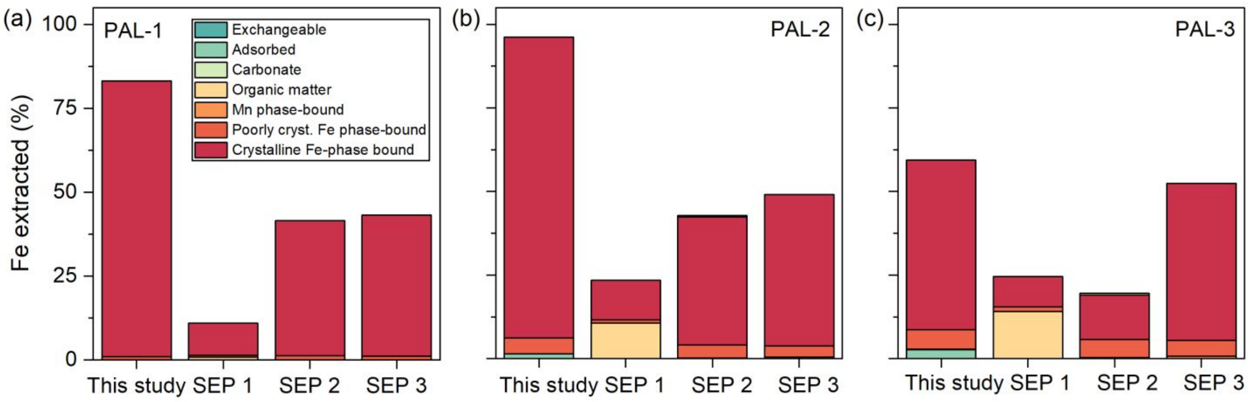

Figure S7. Distribution of Fe (% of total concentration) among the different fractions of the Palawan Ni laterites (PAL-1 – limonite, PAL-2 – transition zone, PAL-3 – saprolite) based on the optimized method (This study) and existing sequential extraction procedures (SEP 1 to 3). The remaining proportion of Cr are distributed in the residual fraction.

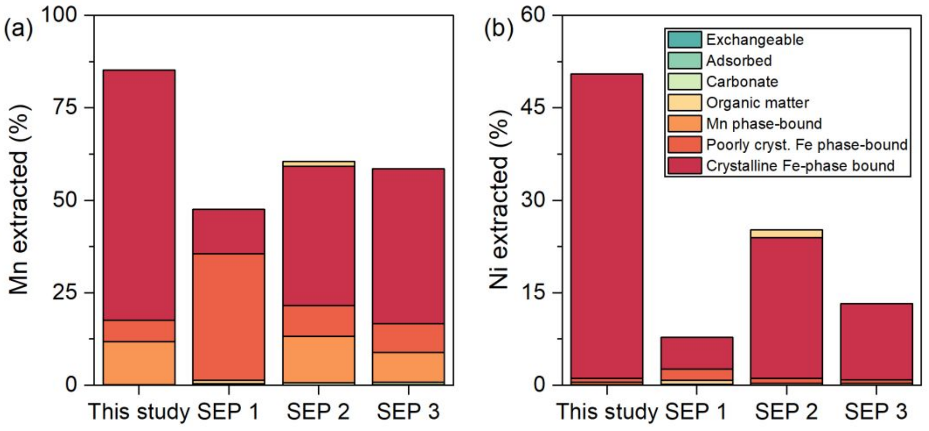

Figure S8. Distribution of extractable (a) manganese and (b) nickel in the Palawan limonite sample (PAL-1) based on the optimized SEP (This study) and existing SEPs.

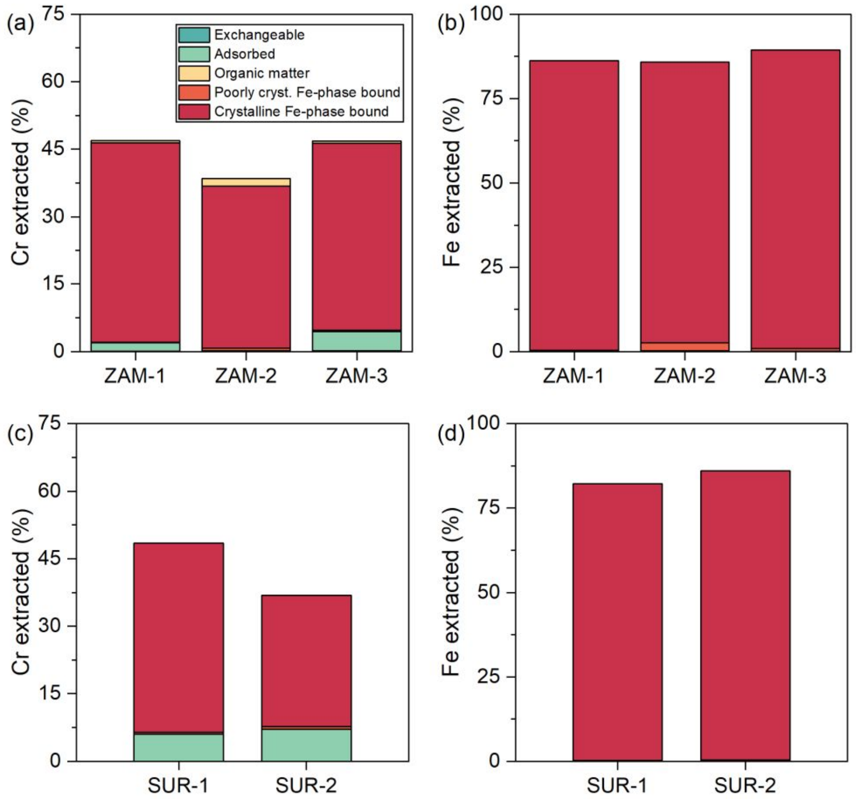

Figure S9. Distribution of Cr and Fe in the (a-b) Zambales (ZAM) and (c-d) Surigao (SUR) limonite samples according to the optimized SEP. The SEP was further simplified by not including the carbonate-bound and Mn-bound fractions, found to host negligible amounts of Cr.

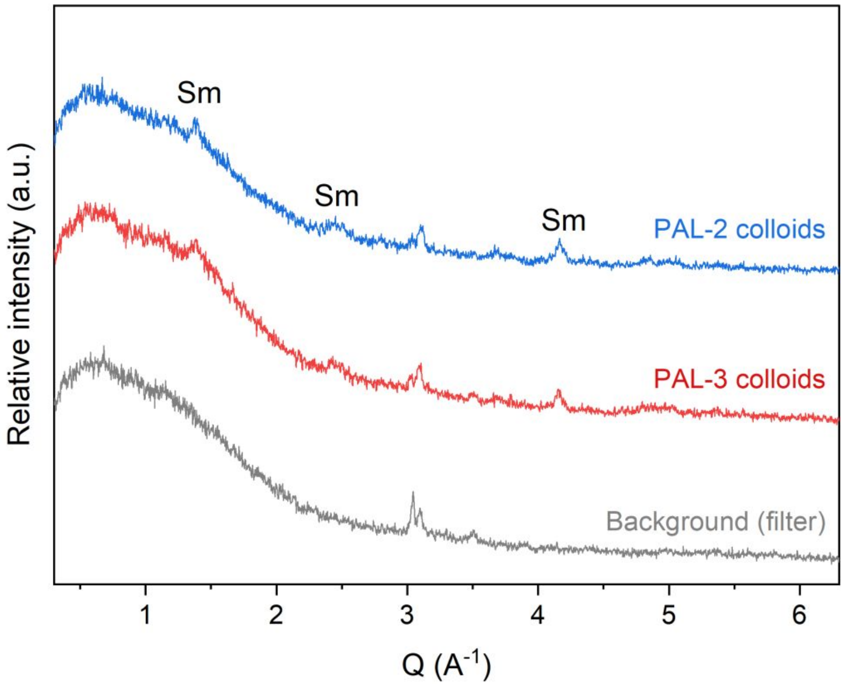

Figure S10. XRD patterns of colloids recovered from vacuum filtration of the phosphate extracted solutions. The thin films of colloids were analyzed with the filter (0.2-μm polycarbonate membrane). Compared with the background signal from the filter, the colloids from PAL-2 and PAL-3 showed diffraction peaks from smectite (Sm).

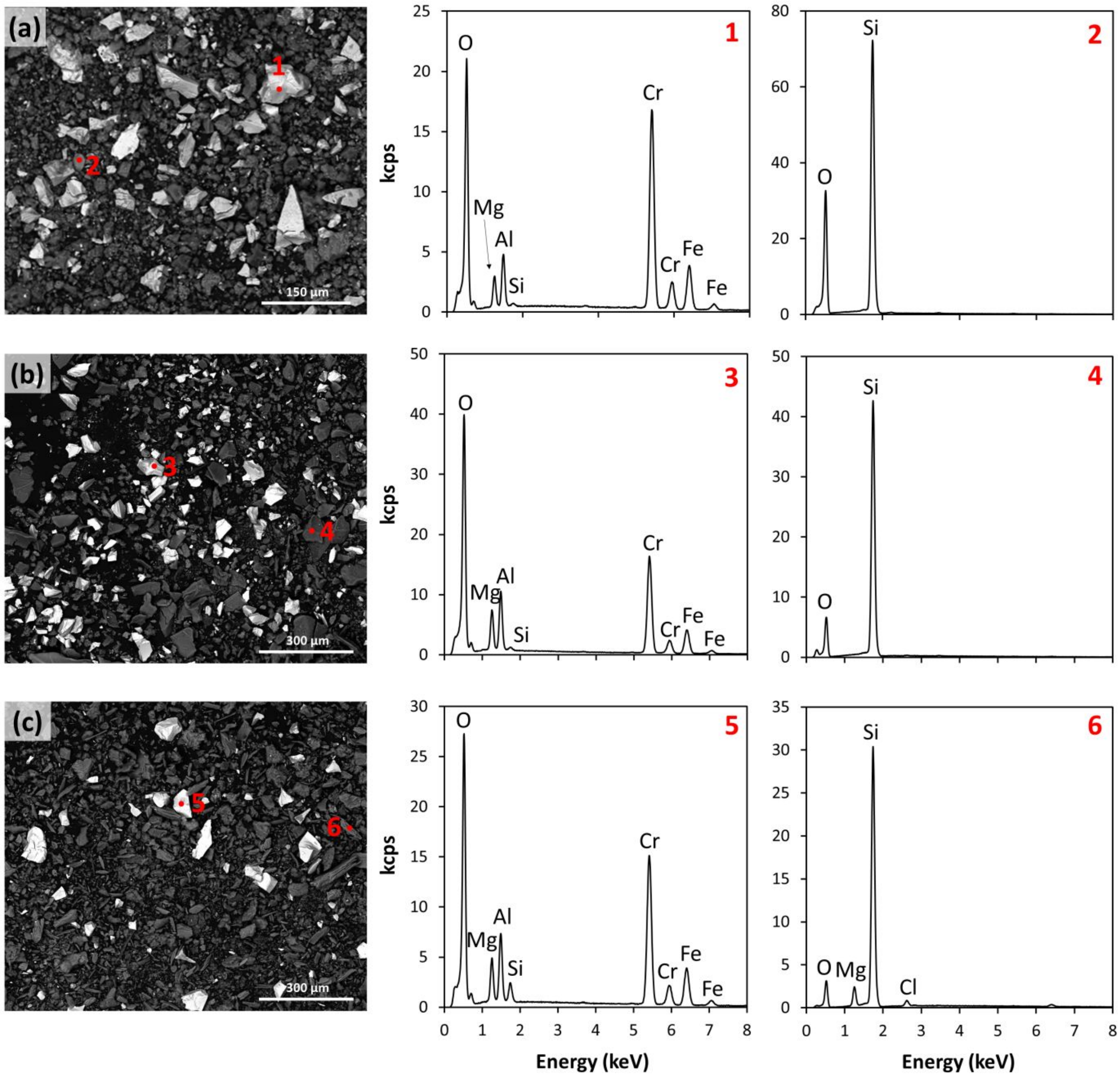

Figure S11. SEM-EDS analyses of chromites and silicate phases of (a) PAL-1, (b) PAL-2, and (c) PAL-3 residues after applying the optimized sequential extraction.

References

1. Toby, B. H.; Von Dreele, R. B., GSAS-II: the genesis of a modern open-source all purpose crystallography software package. *Journal of Applied Crystallography* **2013,** *46*, (2), 544-549.

2. Bokhari, S. N. H.; Meisel, T. C., Method Development and Optimisation of Sodium Peroxide Sintering for Geological Samples. *Geostandards and Geoanalytical Research* **2017,** *41*, (2), 181-195.

3. Becquer, T.; Pétard, J.; Duwig, C.; Bourdon, E.; Moreau, R.; Herbillon, A. J., Mineralogical, chemical and charge properties of Geric Ferralsols from New Caledonia. *Geoderma* **2001,** *103*, (3), 291-306.

4. Fandeur, D.; Juillot, F.; Morin, G.; Olivi, L.; Cognigni, A.; Ambrosi, J. P.; Guyot, F.; Fritsch, E., Synchrotron-based speciation of chromium in an Oxisol from New Caledonia: Importance of secondary Fe-oxyhydroxides. *American Mineralogist* **2009,** *94*, (5-6), 710-719.

5. Schwertmann, U.; Cornell, R. M., *Iron Oxides in the Laboratory: Preparation and Characterization*. Wiley: Germany, 2000; p 204.

6. Bousserrhine, N.; Gasser, U. G.; Jeanroy, E.; Berthelin, J., Bacterial and Chemical Reductive Dissolution of Mn-, Co-, Cr-, and Al-Substituted Goethites. *Geomicrobiology Journal* **1999,** *16*, (3), 245-258.

7. Liu, F.; Li, X.; Sheng, A.; Shang, J.; Wang, Z.; Liu, J., Kinetics and Mechanisms of Protein Adsorption and Conformational Change on Hematite Particles. *Environmental Science & Technology* **2019,** *53*, (17), 10157-10165.

8. Frierdich, A. J.; Luo, Y.; Catalano, J. G., Trace element cycling through iron oxide minerals during redox-driven dynamic recrystallization. *Geology* **2011,** *39*, (11), 1083-1086.

9. Jackson, M. L. R., *Soil Chemical Analysis: Advanced Course*. M.L., Jackson: 1969.

10. Myagkiy, A.; Truche, L.; Cathelineau, M.; Golfier, F., Revealing the conditions of Ni mineralization in the laterite profiles of New Caledonia: Insights from reactive geochemical transport modelling. *Chemical Geology* **2017,** *466*, 274-284.

11. Rai, D.; Eary, L. E.; Zachara, J. M., Environmental chemistry of chromium. *Sci Total Environ* **1989,** *86*, (1-2), 15-23.

12. Bartlett, R. J., Chromium cycling in soils and water: links, gaps, and methods. *Environmental Health Perspectives* **1991,** *92*, 17-24.

13. Keon, N. E.; Swartz, C. H.; Brabander, D. J.; Harvey, C.; Hemond, H. F., Validation of an Arsenic Sequential Extraction Method for Evaluating Mobility in Sediments. *Environmental Science & Technology* **2001,** *35*, (13), 2778-2784.
14. Larios, R.; Fernandez-Martinez, R.; Rucandio, I., Comparison of three sequential extraction procedures for fractionation of arsenic from highly polluted mining sediments. *Anal Bioanal Chem* **2012,** *402*, (9), 2909-21.
15. Wright, M. T.; Parker, D. R.; Amrhein, C., Critical Evaluation of the Ability of Sequential Extraction Procedures To Quantify Discrete Forms of Selenium in Sediments and Soils. *Environmental Science & Technology* **2003,** *37*, (20), 4709-4716.
16. Fandeur, D.; Juillot, F.; Morin, G.; Olivi, L.; Cognigni, A.; Webb, S. M.; Ambrosi, J.-P.; Fritsch, E.; Guyot, F.; Brown, J. G. E., XANES Evidence for Oxidation of Cr(III) to Cr(VI) by Mn-Oxides in a Lateritic Regolith Developed on Serpentinized Ultramafic Rocks of New Caledonia. *Environmental Science & Technology* **2009,** *43*, (19), 7384-7390.
17. Gunkel-Grillon, P.; Laporte-Magoni, C.; Lemestre, M.; Bazire, N., Toxic chromium release from nickel mining sediments in surface waters, New Caledonia. *Environmental Chemistry Letters* **2014,** *12*, (4), 511-516.
18. Gleyzes, C.; Tellier, S.; Astruc, M., Fractionation studies of trace elements in contaminated soils and sediments: a review of sequential extraction procedures. *TrAC Trends in Analytical Chemistry* **2002,** *21*, (6), 451-467.
19. Hass, A.; Fine, P., Sequential Selective Extraction Procedures for the Study of Heavy Metals in Soils, Sediments, and Waste Materials—a Critical Review. *Critical Reviews in Environmental Science and Technology* **2010,** *40*, (5), 365-399.
20. Bartlett, R. J.; Kimble, J. M., Behavior of Chromium in Soils: II. Hexavalent Forms. *Journal of Environmental Quality* **1976,** *5*, (4), 383-386.
21. Oze, C.; Fendorf, S.; Bird, D. K.; Coleman, R. G., Chromium Geochemistry of Serpentine Soils. *International Geology Review* **2004,** *46*, (2), 97-126.
22. Drahota, P.; Grosslova, Z.; Kindlova, H., Selectivity assessment of an arsenic sequential extraction procedure for evaluating mobility in mine wastes. *Anal Chim Acta* **2014,** *839*, 34-43.
23. Tokunaga, T. K.; Lipton, D. S.; Benson, S. M.; Yee, A. W.; Oldfather, J. M.; Duckart, E. C.; Johannis, P. W.; Halvorsen, K. E., Soil selenium fractionation, depth profiles and time trends in a vegetated site at Kesterson Reservoir. *Water, Air, and Soil Pollution* **1991,** *57*, (1), 31-41.

24. Barlett, R. J.; James, B. R., Chromium. In *Methods of Soil Analysis: Part 3 Chemical Methods*, D.L. Sparks, A. L. P., P.A. Helmke, R.H. Loeppert, P. N. Soltanpour, M. A. Tabatabai, C. T. Johnston, M. E. Sumner, Ed. Soil Science Society of America, Inc., American Society of Agronomy, Inc.: USA, 1996; pp 683–701.

25. Perez, J. P. H.; Tobler, D. J.; Thomas, A. N.; Freeman, H. M.; Dideriksen, K.; Radnik, J.; Benning, L. G., Adsorption and Reduction of Arsenate during the Fe2+-Induced Transformation of Ferrihydrite. *ACS Earth and Space Chemistry* **2019,** *3*, (6), 884-894.

26. Scheinost, A. C.; Claussner, J.; Exner, J.; Feig, M.; Findeisen, S.; Hennig, C.; Kvashnina, K. O.; Naudet, D.; Prieur, D.; Rossberg, A.; Schmidt, M.; Qiu, C. R.; Colomp, P.; Cohen, C.; Dettona, E.; Dyadkin, V.; Stumpf, T., ROBL-II at ESRF: a synchrotron toolbox for actinide research. *J Synchrotron Radiat* **2021,** *28*, 333-349.

27. Proux, O.; Lahera, E.; Del Net, W.; Kieffer, I.; Rovezzi, M.; Testemale, D.; Irar, M.; Thomas, S.; Aguilar-Tapia, A.; Bazarkina, E. F.; Prat, A.; Tella, M.; Auffan, M.; Rose, J.; Hazemann, J.-L., High-Energy Resolution Fluorescence Detected X-Ray Absorption Spectroscopy: A Powerful New Structural Tool in Environmental Biogeochemistry Sciences. *Journal of Environmental Quality* **2017,** *46*, (6), 1146-1157.

28. Kvashnina, K. O.; Scheinost, A. C., A Johann-type X-ray emission spectrometer at the Rossendorf beamline. *Journal of Synchrotron Radiation* **2016,** *23*, (Pt 3), 836-841.

29. Ravel, B.; Newville, M., ATHENA, ARTEMIS, HEPHAESTUS: data analysis for X-ray absorption spectroscopy using IFEFFIT. *J Synchrotron Radiat* **2005,** *12*, 537-541.

30. Webb, S. M., SIXpack: a graphical user interface for XAS analysis using IFEFFIT. *Physica Scripta* **2005,** *2005*, (T115), 1011.

31. Newville, M., IFEFFIT : interactive XAFS analysis and FEFF fitting. *J Synchrotron Radiat* **2001,** *8*, (2), 322-324.

32. Rehr, J. J.; Albers, R. C.; Zabinsky, S. I., High-order multiple-scattering calculations of x-ray-absorption fine structure. *Phys Rev Lett* **1992,** *69*, (23), 3397-3400.

33. Lenaz, D.; Logvinova, A. M.; Princivalle, F.; Sobolev, N. V., Structural parameters of chromite included in diamond and kimberlites from Siberia: A new tool for discriminating ultramafic source. *American Mineralogist* **2009,** *94*, (7), 1067-1070.

34. N. Thomas, A.; Eiche, E.; Göttlicher, J.; Steininger, R.; G. Benning, L.; M. Freeman, H.; Dideriksen, K.; Neumann, T., Products of Hexavalent Chromium Reduction by Green Rust Sodium Sulfate and Associated Reaction Mechanisms. *Soil Systems* **2018,** *2*, (4), 58.

35. Bots, P.; Shaw, S.; Law, G. T.; Marshall, T. A.; Mosselmans, J. F.; Morris, K., Controls on the Fate and Speciation of Np(V) During Iron (Oxyhydr)oxide Crystallization. *Environ Sci Technol* **2016,** *50*, (7), 3382-90.

36. Marshall, T. A.; Morris, K.; Law, G. T.; Livens, F. R.; Mosselmans, J. F.; Bots, P.; Shaw, S., Incorporation of Uranium into Hematite during crystallization from ferrihydrite. *Environ Sci Technol* **2014,** *48*, (7), 3724-31.

37. Singh, B.; Sherman, D. M.; Gilkes, R. J.; Wells, M. A.; Mosselmans, J. F. W., Incorporation of Cr, Mn and Ni into goethite (α-FeOOH): mechanism from extended X-ray absorption fine structure spectroscopy. *Clay Minerals* **2002,** *37*, (4), 639-649.

38. Toner, B. M.; Santelli, C. M.; Marcus, M. A.; Wirth, R.; Chan, C. S.; McCollom, T.; Bach, W.; Edwards, K. J., Biogenic iron oxyhydroxide formation at mid-ocean ridge hydrothermal vents: Juan de Fuca Ridge. *Geochimica et Cosmochimica Acta* **2009,** *73*, (2), 388-403.

39. Galivarapu, J. K.; Kumar, D.; Banerjee, A.; Sathe, V.; Aquilanti, G.; Rath, C., Effect of size reduction on cation distribution and magnetic transitions in CoCr2O4 multiferroic: EXAFS, magnetic and diffused neutron scattering measurements. *RSC Advances* **2016,** *6*, (68), 63809-63819.

40. Peterson, M. L.; Brown, G. E.; Parks, G. A.; Stein, C. L., Differential redox and sorption of Cr (III/VI) on natural silicate and oxide minerals: EXAFS and XANES results. *Geochimica et Cosmochimica Acta* **1997,** *61*, (16), 3399-3412.

41. Kelly, S. D.; Hesterberg, D.; Ravel, B., Analysis of Soils and Minerals Using X-ray Absorption Spectroscopy. In *Methods of Soil Analysis Part 5—Mineralogical Methods*, A. L. Ulery, L. R. D., Ed. Soil Science Society of America, Inc.: USA, 2008; pp 387-463.

42. Börsig, N.; Scheinost, A. C.; Shaw, S.; Schild, D.; Neumann, T. A., Uptake mechanisms of selenium oxyanions during the ferrihydrite-hematite recrystallization. *Geochimica et Cosmochimica Acta* **2017,** *206*, 236-253.

43. Benitez, L. N.; Dubois, J.-P., Evaluation of the Selectivity of Sequential Extraction Procedures Applied to the Speciation of Cadmium in Soils. *International Journal of Environmental Analytical Chemistry* **1999,** *74*, (1-4), 289-303.

44. Quantin, C.; Becquer, T.; Rouiller, J.; Berthelin, J., Redistribution of Metals in a New Caledonia Ferralsol After Microbial Weathering. *Soil Science Society of America Journal* **2002,** *66*, (6), 1797-1804.

45. Silveira, M. L.; Alleoni, L. R.; O'Connor, G. A.; Chang, A. C., Heavy metal sequential extraction methods--a modification for tropical soils. *Chemosphere* **2006,** *64*, (11), 1929-38.
